# Maritime Hunter-Gatherers Adopt Cultivation at the Farming Extreme of Northern Europe 5000 Years Ago

**DOI:** 10.1038/s41598-019-41293-z

**Published:** 2019-03-18

**Authors:** Santeri Vanhanen, Stefan Gustafsson, Håkan Ranheden, Niclas Björck, Marianna Kemell, Volker Heyd

**Affiliations:** 1 Archaeology, Department of Cultures, University of Helsinki, P.O. Box 59, Unioninkatu 38, Helsinki, 00014 Finland; 2 Arkeologikonsult, Optimusvägen 14, Upplands Väsby, 134 94 Sweden; 3 Arkeologerna, Statens historiska museer, Instrumentvägen 19, Hägersten, 126 53 Sweden; 4 Arkeologerna, Statens historiska museer, Hållnäsgatan 11, Uppsala, 752 28 Sweden; 5 Department of Chemistry, University of Helsinki, 00014 Helsinki, Finland

## Abstract

The dynamics of the origins and spread of farming are globally debated in anthropology and archaeology. Lately, numerous aDNA studies have turned the tide in favour of migrations, leaving only a few cases in Neolithic Europe where hunter-gatherers might have adopted agriculture. It is thus widely accepted that agriculture was expanding to its northern extreme in Sweden c. 4000 BC by migrating Funnel Beaker Culture (FBC) farmers. This was followed by intense contacts with local hunter-gatherers, leading to the development of the Pitted Ware Culture (PWC), who nonetheless relied on maritime prey. Here, we present archaeobotanical remains from Sweden and the Åland archipelago (Finland) showing that PWC used free-threshing barley and hulled and free-threshing wheat from c. 3300 BC. We suggest that these hunter-gatherers adopted cultivation from FBC farmers and brought it to islands beyond the 60th parallel north. Based on directly dated grains, land areas suitable for cultivation, and absence of signs of exchange with FBC in Sweden, we argue that PWC cultivated crops in Åland. While we have isotopic and lipid-biomarker proof that their main subsistence was still hunting/fishing/gathering, we argue small-scale cereal use was intended for ritual feasts, when cereal products could have been consumed with pork.

## Introduction

**Figure 1:**
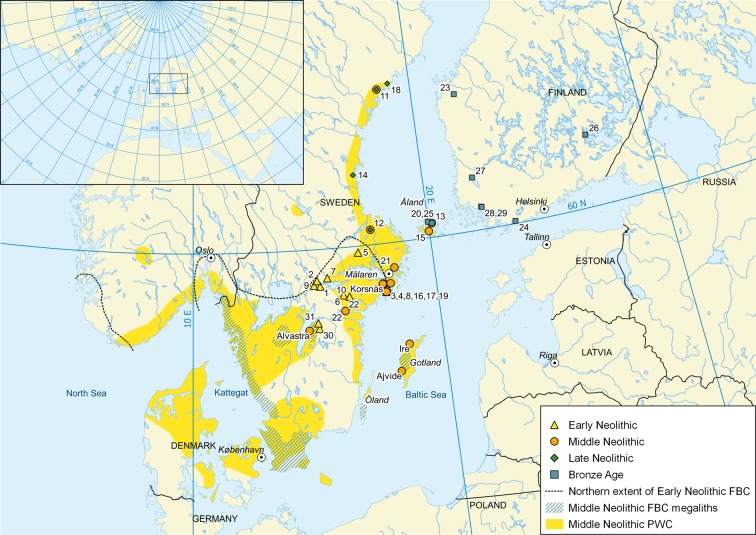
Geographical setting of the study area in northern Europe and location of the sites. Distribution of selected archaeological cultures in northern Europe during the Neolithic period^8,25,34^. Middle Neolithic Pitted Ware Culture (PWC), c. 3500–2300 BC, northern extent of the Early Neolithic Funnel Beaker Culture (FBC), c. 4000–3300 BC, and Middle Neolithic Funnel Beaker Culture megaliths in Sweden, c. 3300–3000 BC. Find locations with numbers demarcate sites where cereal grains have been found and later AMS radiocarbon dated. Cereal grain dates collected NE of Alvastra. More detailed maps provided in Supplementary Figs 1–4. Figure designed by SV and VH. Figure was created by SV using QGIS 3.4. (https://www.qgis.org/) and Natural Earth data (https://www.naturalearthdata.com/).

The first FBC farmers reached the northernmost extreme of farming in east-central Sweden c. 4000 BC, when terrestrial pollen records show high summer temperatures for northeast Europe and Finland (Fig. 1)^1,2^. Here, these farmers founded a regional cluster of settlements during the local Early Neolithic (EN) period, c. 4000–3300 BC, and established a mixed-farming economy by cultivating crops using manure^3^, keeping domestic animals^4^, and consuming milk products^5^. Further to the north, Mesolithic hunter-gathering practices persisted, while to the east both western Finland and the Åland Islands were occupied by Comb Ceramic hunter-gatherers. However, indications of farming and population decline have been found in east-central Sweden at the end of the EN^6^, possibly caused by cooling summer temperatures^7^. FBC moved southwards from east-central Sweden shifting the northernmost border of farming and was replaced here by the PWC c. 3300 BC^8^.

The origins of the PWC are controversial. In one likely scenario, Comb Ceramic^9^ and Mesolithic hunter-gatherers first interacted with FBC during the last centuries of the EN and became specialized maritime hunter-gatherers. The PWC pushed south and westwards during the Middle Neolithic (MN), c. 3300–2300 BC, along the northern Baltic shoreline and adjacent islands, eventually reaching as far west as Denmark and southern Norway^10^. Around 2800 BC, after the FBC ceased to exist, the Corded Ware Culture (CWC) migrated into the PWC area. The end date for the PWC and CWC is approximately 2300 BC, when the material culture was replaced by the Late Neolithic (LN) culture^9^. Spanning nearly a millennium virtually unchanged, the PWC maintained a coherent society and a successful economic model. PWC people lived in marine-oriented settlements, commonly dwelled in huts and produced relatively large amounts of ceramic vessels. This speaks to the partly sedentary nature of their habitation, at least for their base camps. These specialist hunter-gatherers obtained the great majority of their subsistence from maritime sources, such as seal, fish, and sea birds^8,11,12^. Considering the amount of bones, sealing was of paramount importance, causing these peoples to be labelled ‘hard-core sealers’ or even the ‘Inuit of the Baltic’^11^.

In contrast to the more geometric motifs of contemporary FBC farmers, the PWC had an animistic cosmography similar to the Mesolithic and Comb Ceramic hunter-gatherers of the Baltic^13,14^. Both FBC and PWC buried their dead in flat inhumation graves with occasional cremations^13,14^. It was only the FBC farmers in southern Scandinavia who raised megalithic graves^8^, whereas only the PWC hunter-gatherers used red ochre in their burials^14^. The PWC burials were mainly egalitarian, though mortuary houses and secondary burials are evidence of complex burial customs^13^. Osteological measurements have shown that the PWC physiologically adapted to the cold climate, as opposed to other contemporaneous groups^15^. This dialectic has also been demonstrated in recent ancient DNA studies, which conclude that PWC hunter-gatherers and FBC farmers had different genetic origins^16,17^, albeit with minor gene exchange^18^. The PWC population in particular had low genetic diversity, which might speak for a relatively small founder group^16,17^. However, the genetic samples all come from the islands of Öland and Gotland and from southern Sweden, not from east-central Sweden and the Åland Islands, where societal background, interactions, and ecology are fundamentally different. In east-central Sweden, archaeology records a mutual interaction and exchange system, in which not only do FBC ideas become visible in the form and decoration of PWC ceramics, but also stock animals, more rarely cattle and sheep but frequently pigs, make an appearance in the PWC people’s settlement sites and burials^10,19^. The question has been rightly raised whether these were wild boars or domesticated pigs, or even domesticated ones become feral, and consequently whether the PWC people stayed hunters, but turned partly terrestrial, or even became part-time pig raisers and not just consumers^19,20^.

During the last Ice Age, the immense weight of the ice sheet caused large areas of northern Europe to sink below sea level. After the ice melted, these areas slowly emerged again from the sea, transforming the environment around the Baltic Sea. On Åland, new islands emerged and were first visited by Comb Ceramic hunter-gatherers in the late 6^th^ millennium BC; however, they bear no traces of FBC farmers^21^. PWC groups two millennia later had to cross approximately 70 kilometres of open sea to reach these numerous, but rather small, islands, no more than 10 kilometres in diameter at the time.

**Figure 2:**
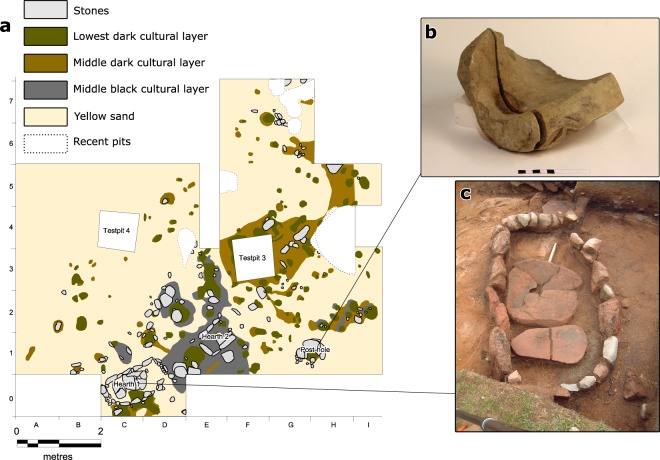
Archaeological finds. (**a**) Excavation plan of the Glamilders site, showing distributions of selected cultural layers, post-holes, and hearths organized in a SW-NE direction. Soil samples were taken from the vicinity of the hearths (sample details in Supplementary Table 5). (**b**) A grinding stone found in a post-hole in Glamilders. (**c**) Hearth 1 from Glamilders, measuring 1.5 × 0.8 metres. Figure a was designed by SV and VH; photos b-c by Jenni Lucenius.

We focus on features and findings from the PWC sites of Glamilders-Svinvallen and Jettböle, located in the Åland islands and dated to c. 3300–2300 cal BC. Excavations have revealed cultural layers, hearths, and post-holes (Fig. 2). Human bones found in occupation layers, special treatment of animal bones, and anthropomorphic clay figurines are examples of ritual activities. We also present material from excavations conducted in east-central Sweden, at the sites of Åby, dated 3250–2300 cal BC, and Tråsättra, dated 2900–2300 cal BC. Here, structures such as huts, hearths, cooking pits, and graves have been discovered (Supplementary Fig. 7).

The diet, animal consumption, and vessel use of the PWC are well understood. However, the PWC’s plant use is poorly known^8^. To study which plants PWC people collected, or whether they even cultivated plants in this pelagic environment, situated at the northern margins of farming, we have studied archaeobotanical plant remains and AMS radiocarbon dated remains from the Åland Islands and east-central Sweden and compared them with earlier and later finds from the region.

## Results

### Spread of cultivation in the northern Baltic

**Figure 3:**
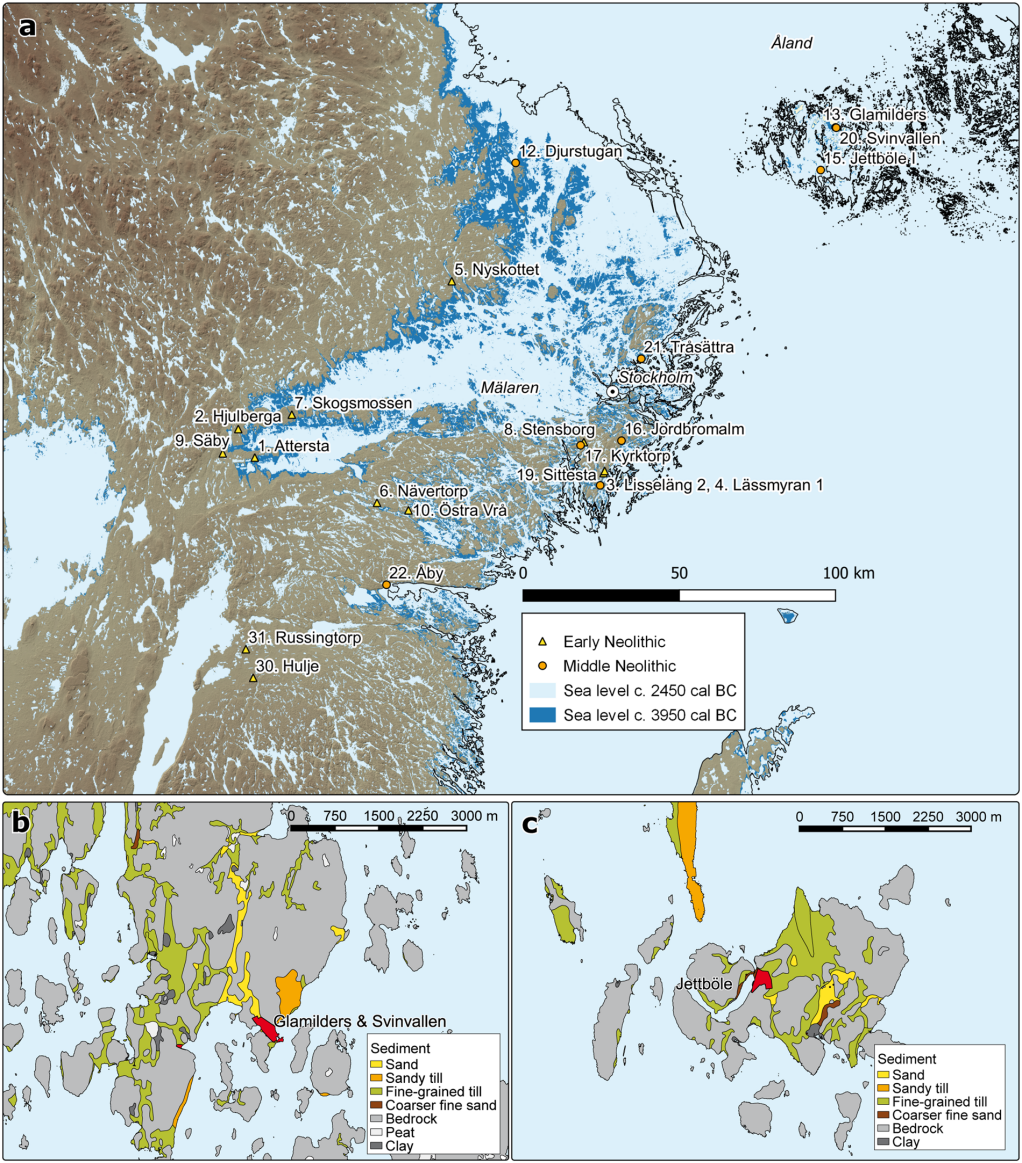
Environmental setting. (**a**) Sites with radiocarbon-dated cereal grains from the EN and MN periods in east-central Sweden and Åland. The sea level is modelled for the time slices of c. 3950 cal BC to 2450 cal BC. The modern coastline is marked with a black line. (**b**) Location of Glamilders and Svinvallen sites, modelled with a sea level of 29 metres above current sea level, marked in red; distribution areas of light sand and light till highlighted. (**c**) Site of Jettböle (I & II), marked in red with a sea level also at 29 metres above the current level, and distribution area of adjacent sand and light till highlighted. Figures were designed by SV and VH. Figures were created by SV using QGIS 3.4. (https://www.qgis.org/) and Natural Earth data (https://www.naturalearthdata.com/).

**Figure 4:**
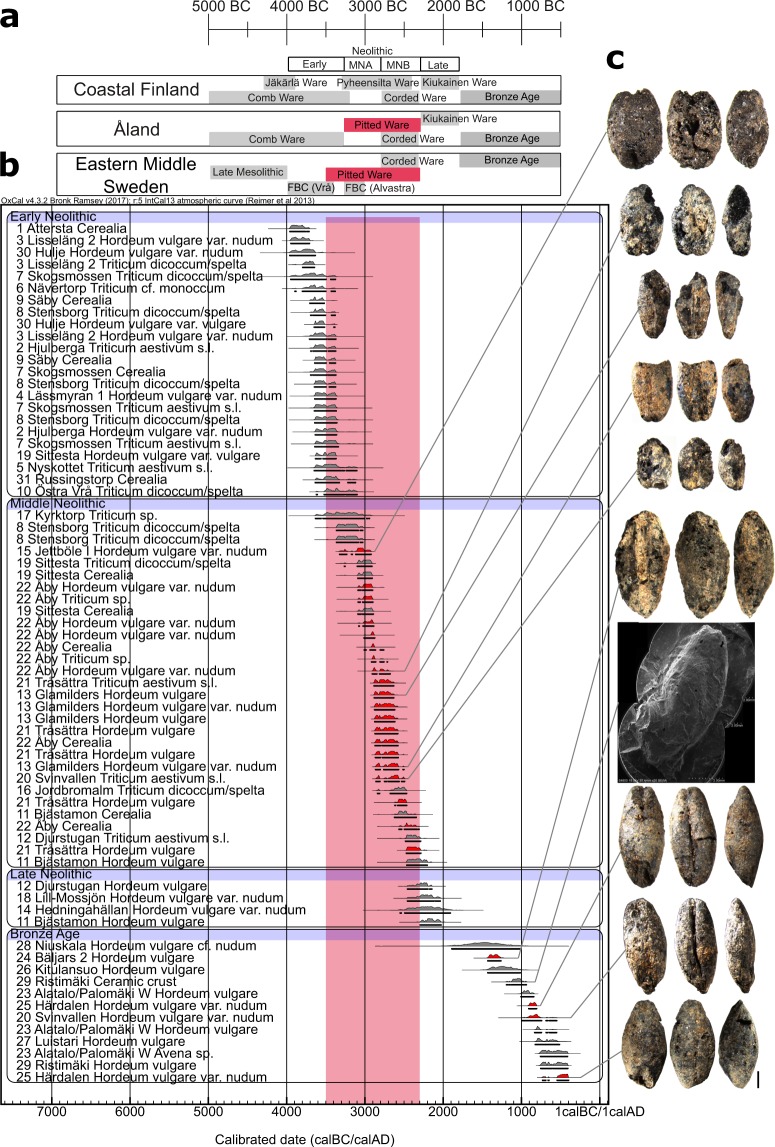
Chronology and radiocarbon-dated cereal grains. (**a**) Chronological scheme of archaeological cultures in different regions of the study area. (**b**) Calibrated AMS radiocarbon dates from cereal grains. New dates presented in the study are highlighted in red, and dates collected from the literature are marked in grey. Bronze Age dates were collected only from Finland and Åland. Site location numbers are in concordance with Figs 1 and 3. For details on the dated cereal grains, see Supplementary Table 2. (**c**) Dorsal, ventral, and lateral views of the grains, and an SEM image of a barley impression. The black scale bar is 1 mm long. Figure designed by SV and VH.

Here, we present new as well as previously published AMS radiocarbon determinations of cereal grains from east-central and northern Sweden, the Åland Islands, and Finland. The radiocarbon dates are discussed in relation to land area at c. 3950 cal BC and 2450 cal BC (Figs 3 and 4).

In east-central Sweden, 23 cereal grains from 13 sites have been AMS radiocarbon dated to the local EN, c. 4000–3300 cal BC. The earliest dated 3950–3700 cal BC and came from the sites Attersta and Lisseläng 2 (Fig. 3). All but one EN cereal grain originated from sites situated south or west of Mälaren (Fig. 3). Cereals have been found at sites situated in inland locations, albeit mostly with access to the sea, and on the large (c. 40 × 40 km) island of Södertörn (Fig. 3: 3–4, 8, 19). No EN cereal grains have been found on the smaller islands in Lake Mälaren. As opposed to the large land areas in east-central Sweden, the Åland archipelago consisted of small rocky islands visited or inhabited by seal-hunting Comb Ceramic groups from the east since 5300 BC^21^. No cultivated plants were discovered at the Comb Ceramic sites of Överby and Kloddberget on Åland dating to c. 5200–3300 cal BC. Wild, possibly gathered, plants found at these sites consist of hazel, copse-bindweed, knotgrass, juniper, and horsetail (Supplementary Results).

In the northern Baltic, 30 cereal grains from 11 sites date to the local MN, c. 3300–2300 cal BC, 20 of which are first presented in this study. Cereal grains have been found south of Lake Mälaren, on Åland, and at the 63^rd^ parallel north along the Norrland coast (Fig. 1: 8, 11–13, 15–16, 19–22). Cereal grains now occur in maritime locations with direct access to the sea (Fig. 3: 13, 15–16, 19–22) and are no longer found at the earlier inland sites west and south of Mälaren (Fig. 3: 1–2, 7, 9). Cereals now also occur on small islands, such as Tråsättra, c. 3 × 4 km wide, Jettböle, c. 3 × 3 km, and Glamilders-Svinvallen, c. 10 × 6 km (Fig. 3b,c). The distribution of cereal grains was clearly more maritime during the MN. We have, however, so far discovered no cultivated plant impressions from CWC, c. 2800–2300 BC, on vessels found in mainland Finland. Instead, the studied sherds had impressions of wild plants: mountain melick, juniper, and wild strawberry (Supplementary Results).

In east-central Sweden and the western Bothnian coast, four cereal grains from four different sites date to the local LN, c. 2300–1900 cal BC. The grains were discovered at coastal sites east and north of Mälaren and along the Norrland coast (Fig. 1: 11–12, 14, 18). Here, we present a new AMS date from Bäljars 2, which is among the oldest radiocarbon-dated cereals in mainland Finland. The earliest radiocarbon-dated cereal grains belong to the LN or Early Bronze Age, c. 1900–1250 cal BC (Fig. 1: 24, 28–29). Additionally, though it is not the focus of this study, we introduce the first cereal grains dated to the Bronze Age from Åland (Fig. 1: 20, 25).

### Cultivated crops and gathered wild plants during the EN and MN periods in the northern Baltic

Thus far, only a limited number of archaeobotanical assemblages dated to the EN and MN have been studied in the region. Nevertheless, some preliminary conclusions can already be drawn based on the species of AMS radiocarbon-dated cereal grains and assemblages of plant remains.

During the EN, c. 4000–3300 cal BC, AMS-dated cereals derived from naked barley, hulled barley, emmer/spelt, naked wheat, and possibly einkorn wheat (Fig. 4). Two assemblages have provided consistent EN dates: the sites of Skogsmossen and Hjulberga (Supplementary Table 3). Barley predominated at Skogsmossen, where naked wheat and emmer have also been found. At Hjulberga, both barley and naked wheat have been found in nearly equal amounts, while only a single emmer grain has been found. Two grains of hulled barley have been dated to the EN, with no larger finds of this crop, making it unclear whether it was intentionally cultivated during the EN. Thus, during the EN three cultivated species have been definitively identified: naked barley, naked wheat, and emmer. We suggest that naked barley was the most common crop, followed by naked wheat and emmer.

The assemblage from the Stensborg site originating during the EN and MN transition contains more than seven thousand cereal grains. The main crop was emmer, followed by naked wheat and lastly by naked barley. Thus, while the cultivation of naked barley, naked wheat, and emmer continued, emmer was now the most common crop, at least at this site.

The cereal grains from the Alvastra site, containing more than nine thousand charred remains altogether, also date to the end of the EN or the MN (Supplementary Table 3). Naked barley was the most common cereal, whereas emmer appears in smaller quantities. At Alvastra, wild gathered plants, such as hazel and crab apple, have also been found.

During the MN, c. 3300–2300 cal BC, AMS-dated cereals consisted of naked barley, naked wheat, and emmer/spelt (Fig. 4). Three site assemblages presented in this study date consistently to this period: Glamilders, Åby, and Tråsättra (Supplementary Table 3). Naked barley is the most commonly identified crop at these sites. Hulled barley was found at Åby and naked wheat both at Åby and Tråsättra. Wild gathered plants occur at all sites too. Hazel is present in each assemblage and was especially common at Glamilders, where we have also found tuber oat grass, crab apple, and rose (Supplementary Fig. 2). At Glamilders and Tråsättra, we have discovered lesser celandine, which was commonly gathered by early agriculturalists in northern Europe^22^. Only a few weed seeds have been found at these sites: false cleavers, cleaver, and knotgrass. The scarcity of weeds might be related to the peculiar harvesting methods, the type of cultivation, or that so few assemblages have been studied.

In addition, naked barley impressions in PWC ceramics from Glamilders date to 2900–2500 cal BC, while wheat impressions from Åby date to the MN, clearly indicating that the predominantly hunter-gatherer PWC people had access to cereals when they were making their ceramics (Supplementary Results).

These discoveries show that naked barley was the main cultivated species during the MN period. Emmer was present at Alvastra, emmer/spelt was at least present at the sites of Sittesta and Jordbromalm, and naked wheat and hulled barley have been found at Åby and Tråsättra, speaking for a diversity in cultivation practices and species.

During the local LN, c. 2300–1700 cal BC, all AMS-dated cereal grains derived from naked barley. However, they represent only small assemblages. During the Bronze Age, c. 1700–500 cal BC, AMS-dated cereal grains on mainland Finland derived from naked barley and now oat, of which oat most probably was not cultivated, but instead was considered a weed during this time. It is difficult to evaluate the importance and presence of different cultivated species during the LN and Bronze Age on mainland Finland due to the overall small quantities of analysed samples; however, it strongly appears that barley was indeed the major crop.

## Discussion

Based on the abovementioned radiocarbon-dated cereal grains from the northern Baltic, there was most likely a regional continuity of cereal cultivation in east-central Sweden during the EN and MN periods, c. 4000–2300 cal BC. Concurrently, we also see a pattern of expansion and suggest three periods for the spread of cultivated plants (Fig. 1). The first spread took place during the EN, when crops appeared around Lake Mälaren. A second spread occurred during the MN, when crops spread further into maritime locations on Åland, the outer islands of Lake Mälaren, and along the Norrland coast. The third expansion occurred during c. 1900–1250 cal BC, when cultivated plants reached eastern parts of mainland Finland.

FBC south and west of Mälaren practiced a mixed farming economy during the EN^5,23^. Though outside the study region, analyses of ancient DNA have recently shown that a FBC female dated to 3945–3647 cal BC (2 sigma) from southern Sweden had genetic affinities with Neolithic farmers in Central Europe^18^, which demonstrates that the spread of FBC in Sweden was, at least partly, due to migration, although local adoption of agriculture by Mesolithic groups has been suggested^24^. Unfortunately, no ancient DNA data are available for the EN in east-central Sweden, but here, as well as in southern Sweden^25^, the change in the material culture can be interpreted as indicating FBC farmer migration combined with interaction with local hunter-gatherer people^23,26^.

In the north-eastern archipelago of Mälaren, we do not find cultivated plants or domestic animals during the EN. While the material culture shows that FBC traits were present^27^, the overall material culture was most strongly affected by hunter-gatherer people of the northern Mesolithic and eastern Comb Ceramic traditions, and it is even possible that local people adopted ceramic manufacture from the FBC farmers^28^. Though cultivation is suggested by finds of cereal-type pollen^29,30^, no macrofossil remains of cultivated plants from the EN have been found on Åland, in Finland, or the Baltic countries^31^.

FBC, Comb Ceramic, and local Mesolithic peoples interacted during the EN in east-central Sweden. Interpretation of archaeological remains suggests that these groups formed a community wherein they transmitted practices requiring intense contacts, as exemplified by ceramic manufacture^9,27^, stone technology^23^, house plans^24^, ritual practices^23^, and even genes^18^. It has been argued that this exchange led to the material expression of the PWC in east-central Sweden from around 3500 cal BC^27^. Maritime subsistence and the hunter-gatherer lifestyle continued after the Mesolithic^28^; however, it appears that small-scale agriculture, suitable for this lifestyle, was inherited from FBC farmers, while PWC users predominantly derived genetically from hunter-gatherers^16–18^.

Former agricultural inland settlements west of Mälaren were abandoned during the Middle Neolithic A (MNA, 3300–2800 BC)^27^, and after this period cereal grains have been found only at coastal sites associated with the PWC (Supplementary Fig. 2). Cereal grain from Åland dated to the period and associated with the PWC shows that this group carried cultivated plants into places without any signs of earlier cultivation. Did the PWC people cultivate domesticated crops themselves, as suggested for western Sweden^32^ or acquire them from the FBC during the MNA?^33^ If we maintain that cereals and other agricultural products were exchanged with other groups, then they must have been brought from the FBC settlements. Such a scenario is unlikely because the FBC retreated from large parts of east-central Sweden and indications of exchange, such as flint imported from southern Swedish FBC to east-central Swedish PWC, greatly diminished^8^. That east-central Sweden was left out of the FBC network is also evident by the lack of megaliths, constructed in southern Sweden mainly during 3300–3000 cal BC (Fig. 1)^34^.

Rachis remains from free-threshing cereals, such as barley, are considered good indications of local cultivation because such cereals were commonly transported as threshed grains^35^. Barley rachis have been discovered at the MN Alvastra site (Supplementary Table 3), which speaks for local cultivation. Three barley rachis fragments have also been discovered at the PWC Sittesta site, with two of them having been found in MN stratigraphical contexts, again indicating local cultivation^36^. Thus far, no threshing remains have been discovered from Åland, but this could reflect the small-scale excavations and the limited number of studied samples. If we, however, hold that cereal grains found at the MNA sites (c. 3300–2800 cal BC) were acquired from the FBC, we must consider that the closest FBC sites of the time were situated in Alvastra and south and west of it (Fig. 1). The distance to Alvastra, associated both with the PWC and FBC, is 100 km from Åby, 200 km from Sittesta, and 370 km from Åland. These distances would make cereal transports, likely in canoes, challenging.

The crops of the earliest north European FBC consisted mainly of naked wheat, barley, and emmer^37^. Around 3750 BC, the focus shifted to emmer and barley^37^. This shift is discernible at the Stensborg and Alvastra sites, where emmer was the most common wheat species (Supplementary Table 3). Naked wheat was, however, still common at PWC sites and it, therefore, appears that PWC adopted crops from the FBC at the beginning of the EN.

During 2800–2300 cal BC, corresponding with the Middle Neolithic B (MNB), cereal grains have been found at coastal locations (Supplementary Fig. 3). Some argue that during the MNB, the PWC in east-central Sweden and Åland acquired cereals and other agricultural products from newcomers of the contemporary CWC^33,38,39^. CWC users inhabiting mainly inland locations in east-central Sweden interacted with the PWC, as demonstrated by the distribution of ceramic finds and similarities in ritual practices^40^. Cultivated plants at CWC sites in Finland were not discovered in the current investigation (Supplementary Results) or earlier studies^41^. In Finland, the keeping of domestic animals is indicated by the evidence of dairy lipids^42^ and mineralized goat hairs^43^. Charred remains and impressions of cultivated plants have been discovered at CWC sites in Estonia^44^ and east-central Sweden^45^ (Fig. 3: 12). In the eastern Baltic region, the earliest bones of domestic animals and a shift in subsistence occurred with the CWC^46,47^. Whether CWC produced the cereals and other agricultural products found at PWC sites is difficult to estimate because only small amounts of plant remains have ever been discovered at CWC sites. The CWC seemingly reached east-central Sweden from regions further to the east^29^, where there is evidence of animal husbandry, but only very few signs of plant cultivation.

Pollen indications of cultivation and animal keeping dated to the MN period have been found in east-central Sweden^48^. Several PWC settlements had sedentary characteristics, as shown by osteological materials from east-central Sweden and Åland^49,50^. These analyses demonstrate that at least parts of the population would have stayed at PWC sites during the crop growing season. Grindstones found from Åland (Fig. 2b) and eastern Sweden could have been used for processing agricultural products. Large accumulations of archaeological finds, pottery in particular, and robust hearth constructions (Fig. 2c) also point towards a substantial sedentary lifestyle, irrespective of the existence of outside camp sites. Small amounts of cattle and sheep bones from PWC sites in east-central Sweden^50^ and on the Åland islands^38^ (Supplementary Materials) further point towards contacts with agricultural groups, or to the small-scale keeping of livestock. Clearly, the Åland islands were too small to maintain a population of wild boars. It is therefore likely that pigs were brought intentionally to the islands by PWC people, though this must be confirmed by direct AMS-dating of the bones^38^.

Overall, arguments for a continuity in cereal grain dates, crop spectra similar to EN FBC, finds of barley rachis, diminished flint exchange between FBC and PWC users, the long distances between FBC and PWC sites, pollen from cultivated cereals in sediment cores, and fully sedentary settlements make it probable that PWC people cultivated their own crops in east-central Sweden and Åland during the MN period. On Åland, the availability and extent of cultivable soils could have been too small for plant cultivation and animal keeping prior to the PWC occupation. Land uplift around 3000 BC, during the occupation of the PWC sites Jettböle and Glamilders-Svinvallen, has revealed light tills and sandy soils, the extent of which permitted small-scale barley and wheat cultivation. Not surprisingly, our PWC sites are located close to them (Fig. 3b,c).

For the Late Neolithic (LN), cereal grains have been found north of Mälaren and along the Norrland coast (Fig. 1). In mainland Finland, the first cereal grains occur during the LN or Bronze Age, c. 1900–1250 cal BC. The earliest bones of sheep/goat from mainland Finland are earlier, dating back to 2200–1950 cal BC^51^. Finds of Scandinavian bronze artefacts indicate an influx from east-central Sweden, which might well be a source area for these agricultural innovations^52^. A similar development is found in the eastern Baltic region, where the earliest directly radiocarbon-dated cereals originate from the Bronze Age, 1392–1123 cal BC (2 sigma)^31^. Thus, agriculture was evident during the Bronze Age in the eastern Baltic, but at least animal keeping and probably crop cultivation were present earlier during the CWC phase.

So far, we have demonstrated that cereals were found at PWC sites in east-central Sweden and on Åland, this being the northern boundary of farming at that time. The cereals were, in all likelihood, cultivated locally by PWC, who might have preferred barley, which is suited to a harsh environment and could have given better yields, and therefore, been naturally selected over wheat species. There is evidence, however, that maritime resources were the main staples of their diet, as shown by finds of animal bones^19^, lipid biomarker analyses^53^, and studies of stable isotopes from human bones^11,12^. If not that important for subsistence, is it possible that cereals were significant for social behaviour, such as feasting.

**Figure 5:**
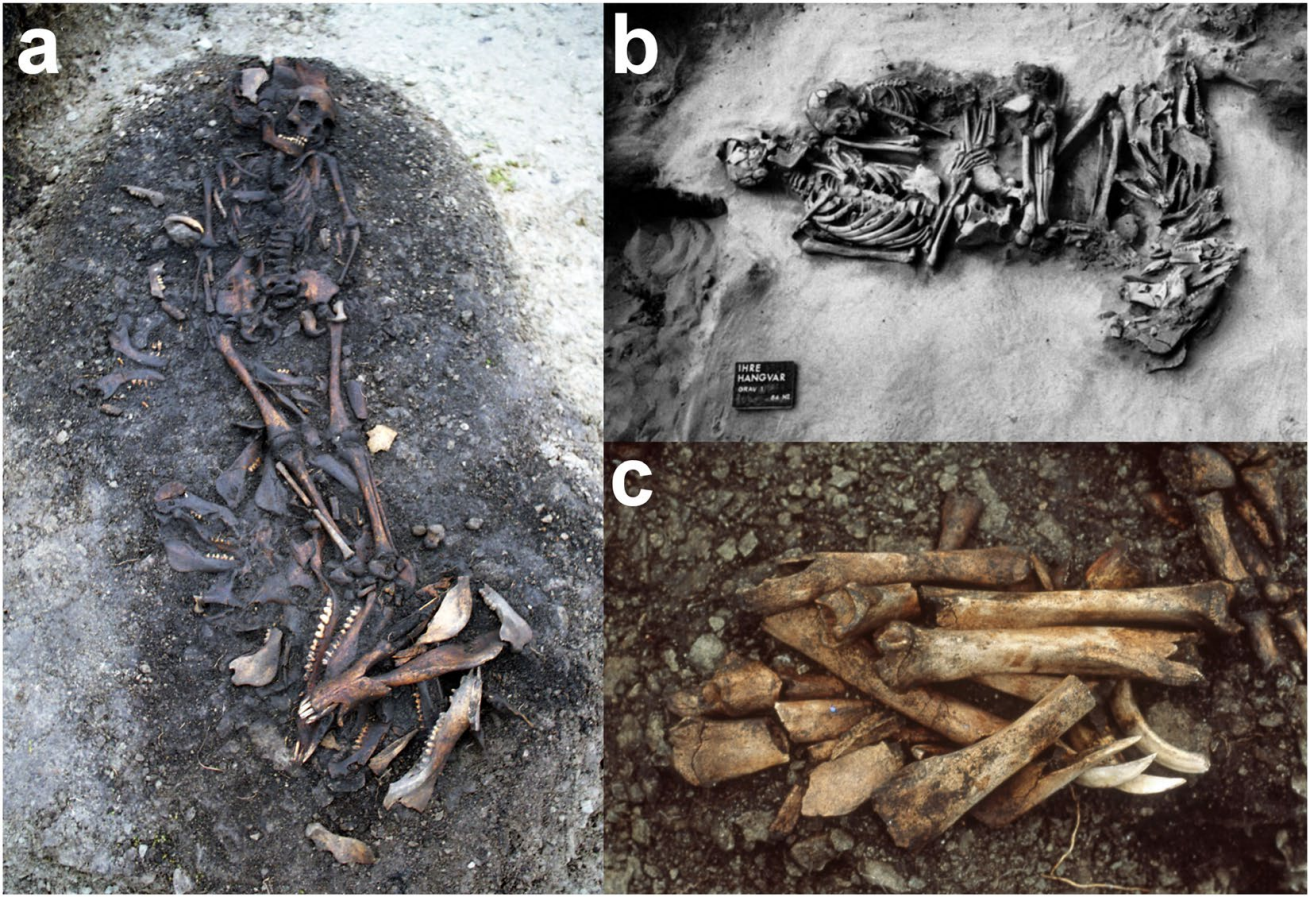
Pig remains in PWC burials on the island of Gotland, Sweden. (**a**) 32 pig jaws deposited at the feet of a seven-year-old child in grave nr. 60 at Ajvide^61^; photo by Göran Burenhult. (**b**) 19 pig jaws laid next to a burial of two adult men, and one 2.5–3-year-old child from grave nr. 7 at the cemetery of Ire^62^; photo by Greta Arwidsson, image from Riksantikvarieämbetet. (**c**) 5 pairs of pig tibia and 4 tusks, deposited in an 18–20-year-old man in grave nr. 6 at Ajvide^61^; photo by Göran Burenhult.

It is common knowledge that communal consumption of food creates social integration and bonding, but also competition, essential for ritual feasts^54^. At feasts, special foods can be consumed; they differ from ordinary meals by having more participants and more food and drink^55^. Feasts could have been an arena for such social integration, bonding, and competition among PWC groups, but also across PWC and FBC. Feasts, in their different forms, have been discussed in numerous historical and ethnographical sources, which can also be used for identifying potential archaeological traces of feasting^55^. Such traces include, for example, unusually large amounts of food remains or food preparation structures accompanied by ritual activity indicators, special features, and human remains^55^. In particular, the large PWC cemeteries discovered on the island of Gotland show numerous features that can be related to ritual feasts. Here, the ritual significance of pigs is most clearly shown by finds of their remains within PWC burials. Up to 32 pig jaws were deposited in a single grave, nr. 7 at the cemetery of Ajvide, and they can be taken to represent the communal consumption of pigs during a feast (Fig. 5)^56^.

Another example of ritual sites associated with the late FBC and early PWC is the remarkable Alvastra pile dwelling (Fig. 1). Here, 100 hearths and numerous fire-making tools, tinder fungus, and numerous prestigious objects have been discovered^8^. At the site, bones from large domestic and wild animals, such as cattle, sheep, pig, and red deer, are common^8^. Alvastra has thus been interpreted as a place for cooking, feasting, cult practices, and assembly^8^.

Fewer graves have been discovered in east-central Sweden and on Åland, probably due to land uplift and acidic soils^14^. At Korsnäs, on Södertörn island (Fig. 1), large amounts of pig bones were discovered dating to the MNA and culturally linked to the PWC^12^. Stable isotopic analyses have indicated that these people had a maritime diet and that pigs were only consumed on special occasions – again, they can best be interpreted as the remains of ritual feasts^12^. Pig bones also occur at several other sites in east-central Sweden and Åland^38,50^, showing that pigs were available in this area.

As for cereals, thousands of their macrofossil remains have been found at Alvastra, and it is possible that they were intentionally burned together with wild plants, such as crab apples^8^. Our cereal finds might thus fall within the context of rare foods to be consumed on special occasions only. Ethnographical studies again provide the best explanation for how domesticated animals and plants that are not used daily were regarded as special food to only be consumed at communally enjoyed feasts^55^. In our case of PWC hunter-gatherers on Åland, who subsisted daily on fat-rich seals, fish, other maritime prey, and gathered plants, the cereals and the pigs were likely such rare specialities. Although not proven in our records, and thus far not tested scientifically, cereals might, however, be converted into alcoholic beverages to accompany rare solid food, thus creating another particular aspect of communal ritual feasting, one well known in ethnography.

## Implications

Our results demonstrate for the first time that domesticated barley and wheat have been found at various PWC sites in east-central Sweden and particularly on the recently enlarging Åland Islands in the Baltic Sea, beyond the 60^th^ parallel north and therefore close to an environmental boundary that even today, with modern technologies, limits cereal farming. Our data confirm these cereals as belonging to contexts spanning this archaeological culture’s entire existence of more than a millennium. However, they also illustrate that PWC hunter-gatherers adopted agricultural practices from their farming FBC neighbours without becoming full-time farmers. Yet, the agricultural practices discussed here are poorly known in detail, and only future investigations will enhance our understanding. Their main subsistence remained throughout their existence as a visible archaeological culture; hunting and gathering the plentifully available resources of the Baltic Sea and its shores. We therefore do not consider the PWC to represent a transitional society whose subsistence economy was on a track towards full-fledged agriculture. Instead, these groups of maritime hunter-gatherers adopted small-scale crop growing as a supplementary skill to produce an additional food resource, likely purposed for special occasions only. Such special occasions were seemingly important enough for a people with hunter-gatherer origins to continue their agricultural practices even in a changing environment, where the donator society of FBC was withdrawing further to the south and away from the PWC hunter-gatherers that are the focus of this study.

## Materials and Methods

The investigations comprised a study of archaeobotanical soil samples, plant impressions in ceramics, archaeological finds, and C14 dates. Soil samples were flotated using mesh sizes of 0.5–0.125 mm. Organic residues resulting from the flotation were studied using a binocular microscope. Discovered plant remains were identified via literature^57^ and reference collections. A complementary method for studying large (20–40 litre) soil samples was used at Tråsättra. This was conducted by first sieving soil through 2 mm sieves and flotating material larger than 2 mm. The aim of the method was to discover rarely occurring cereal grains by expanding the sample volume.

Samples from Kloddberget, Överby, Jettböle I, Jettböle II, Glamilders, Svinvallen, Tengo Nyåker, Kauhala Oxhaga, Bäljars 2, Härdalen, and Ristimäki were studied by SV. Samples from Åby were studied by SG, and samples from Tråsättra by HR. Roger Engelmark analysed six samples from Glamilders excavated in 2004 (ÅM 726): samples 13–15 and 17–18 (Supplementary Table 5).

Plant impressions in ceramics were studied by inspecting the sherds using the naked eye. Promising cavities were studied with a binocular microscope. Dental silicone was used for making casts of the promising cavities. All the casts were then studied with a binocular microscope and compared with modern seed reference material at the Finnish Museum of Natural History. Selected casts were photographed using a camera attached to the binocular microscope, and a proportion of casts were studied using SEM. The SEM images were acquired with a Hitachi S-4800 field emission scanning electron microscope. The specimens were coated with a thin layer of Au-Pd alloy prior to imaging.

Excavation reports were accessed from the archives of Åland Museum in Mariehamn and the Finnish National Board of Antiquities in Helsinki. C14 dates and information from the sites are presented in Supplementary Table 1. Archaeobotanical material was obtained from 13 sites in southern Finland, Åland, and east-central Sweden. The current study includes the study of 220 soil samples with a total volume exceeding 435 litres (the volume of all samples was not recorded). In addition, 9 samples with a total soil volume exceeding 225 litres from Tråsättra were studied using a 2 mm mesh. AMS radiocarbon dating was conducted for 25 plant remains. Plant impressions were studied from CWC materials from southern Finland and preliminarily from sites in Åland. Altogether, 150 casts were made of the CWC material. Details of this material are presented in Supplementary Table 11.

AMS radiocarbon-dated cereal grains are shown in Supplementary Table 2. The dates were calibrated using OxCal 4.3.2^58^ with an IntCal 13 atmospheric curve^59^. The calibrated dates were categorized into the archaeological periods according to their median calibrated value. The aim was to include all dates from east-central Sweden and the Norrland coast prior to c. 2300 cal BC. For Åland and Finland, all dates prior to the Common Era were included. Cereal grain dates or archaeobotanical assemblages from east-central Sweden dating after the MN period were not systematically collected for this study. Archaeobotanical assemblages consisting of charred plants from selected sites with consistent dates are depicted in Supplementary Table 3.

The elevation model presented in Fig. 3a was produced using QGIS software and Copernicus data and information funded by the European Union – EU-DEM layers. For Fig. 3b and c, a digital elevation model of 2 m from the National Land Survey of Finland was used. The site subsoils in Fig. 3b and c were obtained from a soil map of 1:20,000 © by the Geological Survey of Finland 2015. In these data, subsoil is described at a depth of one metre. The shoreline models for Sweden in Fig. 3a were created by the Geological Survey of Sweden. In Fig. 3a, levels of 45 (c. 3950 cal BC) and 34 masl (c. 2450 cal BC) were chosen for Åland based on a recent shoreline study^60^. In Fig. 3b and c, 29 masl was chosen because the sites of Jettböle, Glamilders, and Svinvallen emerge from the sea at this elevation. Radiocarbon dates from Jettböle show that it was first occupied already c. 3300 cal BC and Glamilders and Svinvallen c. 2800 cal BC (Supplementary Table 1).

## Supplementary information

Supplementary Materials for the article: Maritime Hunter-Gatherers Adopt Cultivation at the Farming Extreme of Northern Europe 5000 Years Ago

## Data Availability

All data generated or analysed during this study are included in this published article (and its Supplementary Information files).
