# Supplementary material for: Maritime Hunter-Gatherers Adopt Cultivation at the Farming Extreme of Northern Europe 5000 Years Ago
Source: Sci Rep. 2019 Mar 18;9:4756. doi: 10.1038/s41598-019-41293-z (PMC6426860; doi:10.1038/s41598-019-41293-z)
Supplement: Supplementary file 1 — Supplementary Materials for the article: Maritime Hunter-Gatherers Adopt Cultivation at the Farming Extreme of Northern Europe 5000 Years Ago [file 41598_2019_41293_MOESM1_ESM.pdf]

Supplementary Materials for the article: Maritime Hunter-Gatherers Adopt Cultivation at the Farming Extreme of Northern Europe 5000 Years Ago

Authors:

Vanhanen, Santeri; * SV[1]

Gustafsson, Stefan; SG[2]

Ranheden, Håkan; HR[3]

Björck, Niclas; NB[3]

Kemell, Marianna; MK[4]

Heyd, Volker: VH[1,5]

[1]Archaeology, Department of Cultures, University of Helsinki,

P.O. Box 59, Unioninkatu 38, 00014 University of Helsinki

Santeri.vanhanen@helsinki.fi

[2]Arkeologikonsult, Optimusvägen 14

S 134 94 Upplands Väsby, Sweden

stefan@arkeologikonsult.se

0046735992180

[3]Arkeologerna, Statens historiska museer,

Hållnäsgatan 11, 752 28 Uppsala, Sweden

[4]Department of Chemistry, University of Helsinki,

FI-00014 Helsinki, Finland.

[5]Department of Archaeology & Anthropology

43 Woodland Road, Bristol, BS8 1UU, United Kingdom

Introduction to supplementary materials

The supplementary results are mainly presented in Supplementary tables 1–11. Radiocarbon dates from the sites studied in this paper are presented in Supplementary table 1. Dated cereals are presented in Supplementary table 2. A compilation of archaeobotanical sites with consistent Early Neolithic and/or Middle Neolithic dates with more than ten plant remains is presented in Supplementary table 3. Detailed results of archaeobotanical analyses from sites published in this article are presented in Supplementary results and Supplementary tables 4–11. Supplementary information on sites is based on published materials and excavation reports from the archives of Ålands Museum in Mariehamn and the Finnish National Board of Antiquities in Helsinki.

Supplementary information on sites

Kloddberget

Kloddberget is currently located in a forested and swampy valley with a subsoil of fine-grained till between two steep N–S-oriented hills with exposed bedrock. It is an Early Comb Ware site, and according to C14 dates, it was occupied c. 5200–4300 BC. Patchy and partly overlapping signs of settlement indicate that the site was visited repeatedly. Around c. 4500 BC (55 masl), the site was situated on an approximately 3×4 km island. Kloddberget was situated in a sheltered bay that opened to the south. Contemporaneous sites are situated on other parts of the island. Small amounts of ceramics, lithics of local origin, and whetstones were found at Kloddberget. Archaeobotanical samples derive from Rudolf Gustavsson's excavations in 2007 (ÅM 737)[1].

Anthracological and archaeobotanical analyses were carried out for three samples by Roger Engelmark[2]. Hearth A contained charcoal from *Juniperus* sp., *Betula,* and *Alnus* and five charred copse-bindweed seeds (*Fallopia dumentorum*). Hearth B contained charcoal from *Juniperus, Alnus,* and *Pinus.*

N/E (WGS84): 60.318685, 20.111137

Typological dating: Early Comb Ware

Present research: archaeobotanical study of 12 soil samples with a total volume of 20.5 litres.

Överby

Överby is situated below a hill of barren bedrock. The site is located in a flat area with sandy subsoil that is mostly devoid of stones. Överby is an Early Comb Ware site, and a C14 date indicates that it was occupied during c. 3900–3600 BC. Repeated use of lithics and heating of stones points towards the site being used for longer periods of time. The site was situated on an approximately 1×1 km island, which belonged to a group of small islands, in 4000 BC (50 masl). Överby was situated in a bay facing southwest, sheltered by a nearby cape and islets. Soil samples derive from a hearth dug into the subsoil. Finds from the hearth consist of lithics, a hammerstone, and bones. Archaeobotanical samples derive from Rudolf Gustavsson's excavations in 2002[3].

N/E (WGS84): 60.141223, 20.002736

Typological dating: Early Comb Ware

Present research: archaeobotanical study of one soil sample with a volume of 0.4 litres.

Jettböle I

Jettböle I[4] is situated at the foot of Jättböle hill, to the NW of the hill. It is located in the same meadow as Jettböle II and the subsoil is sand (Fig. 3). Jettböle I is a Pitted Ware site that includes Corded Ware ceramics, and it has been C14 dated to c. 3490–1450 BC. Dates are mainly concentrated in the Pitted Ware period c. 3300–2500 BC. However, an inhumation grave dates back to the Late Neolithic/Bronze Age (1680–1450 cal BC). Site stratigraphy is complicated by burials, pits, and later disturbances[5–7]. Around c. 3000 BC (39 masl), Jettböle I was situated on the east side of an approximately 300 m wide strait opening towards the north on a 3×2 km island. Large areas of fine-grained till and sand were exposed on the island. Jettböle I has been excavated on various occasions. Structures consisted of rectangular hearths, pit houses, and an inhumation grave. Archaeological finds consisted of ceramics, lithics, animal bones, and scattered human remains. Archaeobotanical material derives from Björn Cederhvarf's excavation in 1905[8] with catalogue number NM 4630:120, found in excavation square III, shown in Storå's Fig. 2[5].

Osteological analysis of material from Jettböle I revealed bone material dominated by seal, of which harp seal and ringed seal were the most common[5]. In addition, porpoise, cattle, elk, large ruminant, pig, hare, and dog bones were identified. An analysis of bird bones from Jettböle I revealed at least 15 different species, which were fowled mainly between early spring and early autumn[9]. Eider (*Somateria mollissima*) was the most common bird, followed by velvet scooter (*Melanitta fusca*). Fish bones include cod, flatfish, and Baltic herring[9].

N/E (WGS84): 60.138214, 19.972619

Typological dating: Early Pitted Ware, Corded Ware

Present research: Identification of plant finds and radiocarbon dating.

Jettböle II

Jettböle II[10] is currently situated in a meadow in a flat agricultural area bordered by rocky hills to the south and west. Plant finds have been retrieved from Trench 1 dating back to the Pitted Ware period, c. 2620–2310 cal BC according to C14 determinations. At around c. 2500 B.C. (34 masl), Jettböle II was situated on the east side of a 300 m wide sheltered strait on a 3×3 km island. Large areas of fine-grained till and sand were exposed on the island. Trench 1 (1 m^2) was excavated in the year 2000, and the rich finds from it consist of ceramics, lithics, burnt clay, clay daub, bones, bone tools, and fragments of clay idols. The lithics consist of an axe preform, a peg, a hammerstone, and a whetstone. The lithics were mostly of local origin, but some imported flint was also present. According to a preliminary osteological analysis, bone material consists mostly of seal together with some fish and bird bones and a single cattle tooth. Archaeobotanical material derives from excavations by Jan Storå et al. in 2000[10].

N/E (WGS84): 60.139365, 19.974252

Typological dating: Late Pitted Ware, Corded Ware

Present research: Study of plant finds

Glamilders

Glamilders is situated on a gently southward-sloping hill on sandy subsoil (Figs. 2, 3). The area was previously cultivated and is currently occupied by a small house. Higher areas above the settlement have bedrock near the surface and are forested. Glamilders is part of a larger settlement area inhabited from the Pitted Ware Culture to the Bronze Age periods. Glamilders is the oldest site bearing Late Pitted Ware

material, and the area excavated in 2004 has been C14 dated to 2950–2650 BC (Supplementary table 1). When Glamilders was settled in c. 2500 BC (34 masl), it was situated on the westernmost parts of two islands separated by a narrow sound, with a total size of 10×6 km. The site was situated on a southward-pointing cape in a sheltered small bay surrounded by a larger bay. Archaeobotanical material derives from excavations by Rauno Vaara in 2004[11].

The samples discussed in this article come from trench A in the lower part of the settlement (Fig. 2). Part of the samples were studied by Engelmark et al.[12] The upper part of the settlement has been excavated at various times during the 20th century. Excavations at Glamilders unearthed intact cultural layers, hearths, house depressions, and postholes. Samples studied from the site originating from the uppermost layer, with recent finds, are not included in this paper. Finds at the site consist of ceramics, lithics, amber, bones, hazelnut shell fragments, and red ochre. Ground stone tools found at the site are numerous and include axes, chisels, a rhomboid-shaped pendant, an awl, whetstones, and a stone with a frog-like relief carving. Flint and amber indicate the presence of imported objects. Partly burned and partly unburned bone material has not been studied in detail, but a preliminary analysis has shown the presence of seals, fish, birds, and possibly also cattle or elk, and pigs.

N/E (WGS84): 60.300650, 20.141178

Typological dating: Late Pitted Ware

Present research: Archaeobotanical study of 12 soil samples with a total volume of 27.7 litres, a study of plant impressions, and radiocarbon dating.

Åby

Åby[13,14] is located on sandy soil on a gentle slope facing southwards. Most of the site has been exploited and built over with houses, roads, and schools. The site is large, probably around 25,000 m^2. It has been known about since the 1920s, and over the years 16 different archaeological surveys and excavations have been conducted at the site. In 2014, a 2800 m^2 area in the central part of the site was excavated. Archaeobotanical material derives from excavations by Helena Andersson in 2013[13] and Henrik Runeson and Britta Kihlstedt in 2014[14].

The Åby site is dominated by Pitted Ware material, and the C14 dates fall between c. 3250 and 2300 cal. BC. The dated material is primarily carbonized seeds and hazelnut shells. The site is situated 26–29 m above sea level, which means that at the time of the settlement, the site was near the seashore, on a low promontory close to the mouth of a narrow bay. More than 50 Pitted Ware sites are currently known about along the coastline east of Åby.

The excavations have yielded many different structures, such as large pits, huts, and graves. In 2014, several large pits with large amounts of pottery, some possible circular hut structures with postholes, and pits with red ochre that we interpret as graves were excavated. However, no human bones were preserved. There seem to be 4–5 areas with a similar set of features — a grave, one or two large pits, and a possible hut. Perhaps these reflect different households.

The archaeological record is dominated by large amounts of pottery (Pitted Ware): 145 kg of pottery was collected in the 2014 excavation, and earlier excavations have resulted in more than 500 kg of pottery. Most of the pottery is of the Fagervik III or Fagervik IV type. Some of the sherds have wear that indicate a secondary use as tools. Also, some animal figurines made of clay were found. The lithics consist of debitage of quartz, quartzite, greenstone, porphyrite, sandstone, and flint and include tools such as greenstone axes

and adzes, some flint points, and slate and grinding stones made of sandstone. Some burnt clay and a few fragments of amber have also been found.

The osteological material consists of approximately 1 kg of heavily fragmented and mainly burnt animal bones (c. 10,000 fragments). Fish bones make up 42% of the material and derive mainly from cod with inclusions of pike, eel, salmon, perch, carp, turbot, and herring. Mammal bones make up 36% of the material and derive mainly from beaver and seal, with inclusions of a few ruminants, marten, boar, and medium carnivores. Unidentifiable bones make up 22% of the material.

N/E (WGS84): 58.663475, 16.177410

Typological dating: Late Pitted Ware

Present research: Archaeobotanical study of 113 soil samples with a total volume of 208 litres, a study of plant impressions, and radiocarbon dating.

Tråsättra

Tråsättra[15] lies on a sandy, gently sloping terrace that is situated 26–33 metres above sea level. South of the site, the slope has a steeper gradient towards a pasture with clayey soil situated approximately 18 metres above sea level. The sandy terrace is delimited towards the east and west by cliffs that sheltered the location. Towards the north, there are also higher forested areas with bedrock near the surface or even exposed bedrock. The area has never been cultivated since its PWC occupation, and therefore, finds and constructions are well preserved. Several Neolithic sites (c. 4000–2300 BC) are situated near Tråsättra. When the site was in use (c. 2900–2300 BC), the sea level was situated at around 27 masl, and the area was rich in islands. Tråsättra was then located in the outer archipelago on an island measuring approximately 3×4 km. The site lay on a southward-pointing cape in a bay sheltered by nearby islands.

At Tråsättra, the whole site was excavated, and in the central area the cultural layer was sieved in its entirety. The samples discussed in the article come from different parts of the site, but mainly from the area around the houses and the central area between these constructions (Supplementary fig. 7). A rich and varied inventory of finds from the site consists of ceramics, lithics, and bones. Hazelnut shells were collected during the excavations. The ceramics consist of pots with a rim diameter of 3–50 centimetres, and a rich variety of clay figurines has also been found depicting humans, seals, bears, pigs, snakes, elks, and birds. The lithics consist of picks, awls, chisels, scrapers, saws, axes, whetstones, arrowheads, knives, and other types of tools. On the site, there are both burned bones and a small number of unburned bones. Osteological analysis has shown that seals and fish bones predominated, but a large array of bird and terrestrial mammal bones have also been found. Terrestrial species consisted of cattle, dogs, pigs, beaver, wild cats, otter, elk, brown bears, mountain hares, red foxes, polecats, red squirrels, and pine marten. Birds included tufted duck, red-breasted merganser, great crested grebe, northern shoveler, Eurasian wigeon, common scoter, grey heron, and common eider. Fish consisted of perch, pike, cod, carp, salmon, pikeperch, cyprinids, herring, whitefish, and roach. Maritime mammals consisted of harp seals, ringed seals, and porpoises.

N/E (WGS84): 59.472128, 18.350873

Typological dating: Pitted Ware

Present research: Archaeobotanical study of soil samples, 46 soil samples in all with a total volume of 138 litres, using a 0.5 mm sieve, and nine samples with a total volume exceeding 225 litres with a 2 mm sieve, a study of plant Impressions, and radiocarbon dating.

Svinvallen

Svinvallen is situated on the same gently sloping sandy hill as Glamilders. The area is currently forested and partly damaged by a sandpit. Svinvallen contains mainly Pitted Ware ceramics. Two radiocarbon dates from Svinvallen (2859–2495 cal BC and 997–551 cal BC) point to a long period of use. During the Pitted Ware phase of Svinvallen (2500 BC, 34 masl), it was situated in the same bay as Glamilders. By around 1000 BC (20 masl), the island had grown much larger and was approximately 12×7 km. The environment had also become much more sheltered. Svinvallen has been excavated at various times, and the soil samples derive from the excavations of 2015. Small-scale excavations revealed a pit, and finds consisted of ceramics, lithics, a whetstone, a clay idol fragment, bones, and hazelnut shell fragments. Both seal and fish bones were identified during a preliminary analysis.

N/E (WGS84): 60.298973, 20.145789

Typological dating: Late Pitted Ware, Kiukainen Ware

Present research: Archaeobotanical study of nine soil samples with a total volume of 40.4 litres and radiocarbon dating.

Tengo Nyåker

Tengo Nyåker is situated on the SE slope of a sandy ridge on a southward-facing small sandy spit. Large parts of the slopes are cultivated. Clay areas lie below the site. The site has been typologically dated to the Corded Ware period. Tengo Nyåker was situated 400 m NW of a lakeshore around c. 2500 BC (25 masl), and the closest seashore was a bay near the site of Kauhala, 2 km to the east.

The majority of the material derives from Aarne Äyräpää's excavation in 1926 (NM 8709)[16] and Torsten Edgren's excavation in 1982 (NM 21501)[17]; the latter excavation was partly unavailable for this study. Structures on the site consist of two hearths, possible postholes, pits, and cultural layers. One of the hearths was possibly used for producing ceramics. Finds from Tengo Nyåker consist of 18 kg of pottery, clay spoon fragments, lithics (axes, adzes and quartz), and a single burned bone.

Plant impressions in ceramics from Edgren's excavation were preliminarily studied by Merja Seppä-Heikka, and the finds from this study include 16 cf. red-berried elder (cf. *Sambucus racemosa*), one greater celandine (*Chelidonium majus*), three indeterminable seeds, and seven stalk impressions[17].

Ancient lipids from ceramic sherds found at Tengo Nyåker have revealed the remains of dairy fats and ruminant carcass fats[18].

N/E (WGS84): 60.220157, 24.449645

Typological dating: Corded Ware

Present research: Archaeobotanical study of plant impressions

Kauhala Oxhaga

Kauhala Oxhaga[19] is situated on the western slope of a sandy NE-running ridge of sandy till. The slope was cultivated during the 20th century and currently is partly forested and partly a meadow. Clay areas are situated at lower elevations surrounding the site. The site has been typologically dated to the Corded Ware

period. Kauhala Oxhaga was situated 300 m NW of the seashore on a sheltered bay at around 2500 BC (25 masl).

Material from Oxhaga has been collected from the fields and nearby forested areas. The finds consists of lithics and Corded Ware ceramics[19].

N/E (WGS84): 60.225012, 24.482172

Typological dating: Corded Ware

Present research: Archaeobotanical study of plant impressions

Bäljars 2

Bäljars 2 is a dwelling and cultivation site situated on a sandy ridge in the NW part of Lake Lepinjärvi[20]. At around c. 300 BC, Bäljars was situated in a sheltered bay with access to the Baltic Sea. The area has been cultivated, but it is now covered with buildings. Various pit features, hearths, and ard marks were discovered during the excavations. The finds consisted of ceramics, flint, quartz, and burnt bones. Ceramics consisted mainly of the Morby type, and ceramic sherds with textile impressions and ceramics with striated surface were also found. Geochemical, archaeobotanical, and anthracological analyses were conducted for samples taken during the excavations. Radiocarbon dates from the site range from the Bronze Age to the Medieval Period (Supplementary table 1). The current dated barley grain derives from context R5[20].

N/E (WGS84): 60.298973, 20.145789

Typological dating: Bronze Age, Early Iron Age

Present research: Radiocarbon dating.

Härdalen

Härdalen consists of 54 burnt mounds situated on the slopes of a rocky hill facing mostly southwards. The mound studied here dates back to the Bronze Age and was built over a Stone Age settlement. C14 dates span the years 900–400 cal BC. The site was situated on a small, 0.5×1 km island until 2000 BC (29 masl), when it became part of a larger, 7×2.5 km island. In 1000 BC (20 masl), the former bay east of Härdalen was cut off from the sea and became the still existing Lake Dalsträsk. A zone of fine-grained till surrounds the hill at lower elevations and the sediment in the surrounding valley bottoms is clay. Finds from the mound consist of ceramics, lithics, bones, burned clay, hazelnut shell fragments, and a piece of bronze chain and a human tooth. Archaeobotanical material derives from excavations done by Maija Nunez in 1990 and 1991[21].

Osteological analysis has revealed the bones of harp seals, ringed seals and porpoises and a human tooth[5]. Bronze-Age levels of the mound contained cattle teeth, a sheep/goat tooth, and a horse tooth. The Pitted Ware layers contained no terrestrial mammals. Bird bones from Härdalen consisted of bones from different Anatids and especially those from the common eider (*Somateria mollissima*)[22].

N/E (WGS84): 60.336582, 19.991964

Typological dating: Pitted Ware, Kiukainen Ware, Comb Ceramics, Bronze Age

Present research: Archaeobotanical study of 12 soil samples, plant finds, and radiocarbon dating.

Ristimäki

Ristimäki[23] is situated in the Aurajoki river valley on a hill approximately 250 m NW of the river. Large areas of clay subsoil are present with patchy rocky areas and areas of sandy till. According to two C14 dates, Ristimäki dates back to 1190–410 cal BC[24]. At around 1000 BC (15 masl), Ristimäki was situated near an estuary and 2 km from the sea. It was thus a sheltered location providing good communication routes. Soil samples and one plant impression on a Kiukainen ware sherd (TYA 863:9) from the site were investigated. Finds from the site consist of possible post holes, lithics, and Kiukainen ceramics. Archaeobotanical material derives from excavations done by Tytti Juhola et al. in 2013[23].

N/E (WGS84): 60.470469, 22.342774

Typological dating: Kiukainen Ware

Present research: Archaeobotanical study of 15 soil samples, a study of plant impressions.

Supplementary results

At Kloddberget, 12 samples from two hearths (A and B) dug into the subsoil were studied (Supplementary table 4). These contained 97 charred plant remains. The older hearth B, dated to 5210–4958 cal BC, contained four juniper seeds (*Juniperus communis*). Species identified in the younger hearth A, dated to 4488–4347 cal BC, included two hazelnut fragments (*Corylus avellana*), 62 knotgrass seeds (*Polygonum aviculare*), five juniper seeds, and two horsetail branch fragments (*Equisetum* sp.).

Macrofossils from Överby consist of juniper remains (*J. communis*): ten seeds, 18 cf. seed fragments, and one needle. One radiocarbon date from charcoal from Överby dates it to the Comb Ceramic period (3905–3637 cal BC).

Archaeological plant finds from Jettböle I contain one naked barley grain (*Hordeum vulgare* var. *nudum*), three cereal fragments (Cerealia), and 30 hazelnut shell fragments (*Corylus avellana*). A naked barley grain from Jettböle I was dated to the Pitted Ware period (3324–2923 cal BC).

Archaeological plant finds from Jettböle II contain ten rose seeds (*Rosa* sp.), nine rose hips (*Rosa* sp.), four rose testa fragments (*Rosa* sp.), and one hazelnut shell fragment (*Corylus avellana*).

Altogether, 262 charred plant remains were found at Glamilders (Supplementary table 5, Supplementary fig 6). Four barley grains from Glamilders were dated to the Pitted Ware period (Supplementary table 2). The most common remains were hazelnuts, of which 177 fragments were found in the soil samples. In addition, during the excavations 420 finds containing hazelnut shells were collected, comprising altogether approximately 850 grams of charred hazelnuts (most often a find contained numerous shell fragments). Other plants gathered from the wild consisted of 20 rose remains (*Rosa* sp.), two crab apple seeds (*Malus sylvestris*), three root tubers of lesser celandine (*Ranunculus ficaria, cf.*), and one false oat grass basal internode (*Arrhenatherum elatius* var. *bulbosum*). Cultivated plants comprised two naked barley grain (*Hordeum vulgare* var. *nudum, cf.*) and one wheat grain (*Triticum* sp.). Two impressions of barley were discovered in a preliminary study of the ceramics (on sherds ÅM 726:2091, 2271). Aquatic species are represented by three bur-reed seeds (*Sparganium erectum, S. natans, Sparganium* sp.). Arable weeds or plants growing on the shoreline are represented by one small water-pepper seed (*Persicaria minor*), two knotgrass seeds (*Polygonum aviculare*), and two false cleaver seeds (*Galium spurium*). One Norway spruce needle (*Picea abies*) from the sample has not been dated (not included in Supplementary table 4), and it might well be a recent intrusion into the sample, which also contains some earthworm cocoons.

A total of 350 charred plant remains were found at the site of Svinvallen (Supplementary table 6, Supplementary fig 6a). Of these remains, 322 were charred hazelnut shells (*Corylus avellana*). One rose (*Rosa* sp.) seed represents another gathered plant from the site. A bread/club wheat grain from Svinvallen was dated to the Pitted Ware period and a naked barley grain to the Bronze Age (Supplementary table 2). Cultivated plants are represented by a bread/club wheat grain (*Triticum aestivum* s.l.) and a naked barley grain (*Hordeum vulgare* var. *nudum*). The sample containing the wheat grain also contained false cleaver seeds (*Galium spurium*) and goosefoot seeds (*Chenopodium* sp.), which could represent arable weeds accompanying grain.

Material from Härdalen consists of 56 charred plant remains (Supplementary table 7). Two naked barley grains from Härdalen were dated to the Bronze Age (Supplementary table 2). The upper levels (3–5) of the burnt mound contained three barley grains (*H. vulgare* var. *nudum, H. vulgare*), one cereal grain, one raspberry seed (*Rubus idaeus*), one grey club-rush seed (*Schoenoplectus tabernaemontani*), one false cleaver seed (*Galium spurium*), and one horsetail branch (*Equisetum* sp.). Naked barley found during the excavations (ÅM 642:116) originates from layer 3 in the W-section of the mound. Lower excavation levels 8–10 contained 41 hazelnuts, one cereal grain, two lady's mantle seeds (*Alchemilla* sp.), three rose seeds (*Rosa* sp.), and one marsh woundwort seed (*Stachys palustris*).

Ninety-seven charred plant remains were found at Åby (Supplementary tables 10–11). The majority of these remains (75) were cereals, of which naked barley, hulled barley, and bread/club wheat were identified. In addition, 18 hazelnut shells, *Trifolium* sp., and *Vicia* sp. were discovered. Six cereal grains from Åby were dated to the Pitted Ware Period (Supplementary table 2). One impression of emmer/spelt wheat (*Triticum dicoccum/spelta*) and one impression of bread/club wheat (*Triticum aestivum* s.l.) were discovered in the ceramics from Åby.

Archaeobotanical material from Tråsättra consisted of 12 charred plant remains; hazelnuts were not quantified (Supplementary table 9). Cultivated plants consisted of barley and bread/club wheat. Wild plants, namely hazel and lesser celandine root tubers, were also found. One possible arable weed, cleaver, was found. Five cereal grains from Åby were dated to the Pitted Ware period (Supplementary table 2).

Corded Ware sherds from Tengo Nyåker and Kauhala Oxhaga contained small numbers of plant impressions (Supplementary table 11, Supplementary fig. 6). Sixty of the identified casts were pieces of small twigs, stalks, or the like, which could not be further identified. There were also nine hair impressions, one impression of a piece of wood, four possible bud impressions, and one impression of a crack in a vessel. The origin of 79 of the casts could not be determined. The identified impressions from Tengo Nyåker contain mountain melick (*Melica nutans*) (NM 8709:29) on the vessel surface and juniper (*Juniperus communis*) (NM 8798:25) and a possible fruit of bird cherry (cf. *Prunus padus*) both inside the clay matrix of the vessel. At Kauhala Oxhaga, a wild strawberry seed (*Fragaria vesca*) (NM 7852:6) impression was discovered on the vessel's surface. Juniper, cf. bird cherry, wild strawberry, and mountain melick might all have been used as wild gathered plants, but this small number of remains should be considered with caution.

A barley grain from Bäljars 2 was dated to the Bronze Age (Supplementary table 2). The same context R5[20] contained six barley grains (*Hordeum vulgare,* cf. *Hordeum vulgare*), two oat grains (*Avena* sp., cf. *Avena* sp.), seven cereal grains (Cerealia), three hazelnut shell fragments, three lesser celandine root tubers (*Ranunculus ficaria*), one juniper seed (*Juniperus communis*), and three spruce needle fragments (*Picea abies*).

From Ristimäki, one charred grain of barley (*Hordeum vulgare*) was C14 dated to the Bronze Age (Supplementary table 2). The organic crust on a sherd from the site dated back to 1190–940 cal BC (TYA 863:9) and contains an impression of barley (*Hordeum vulgare*) (Supplementary fig. 6e-f).

Supplementary Figures

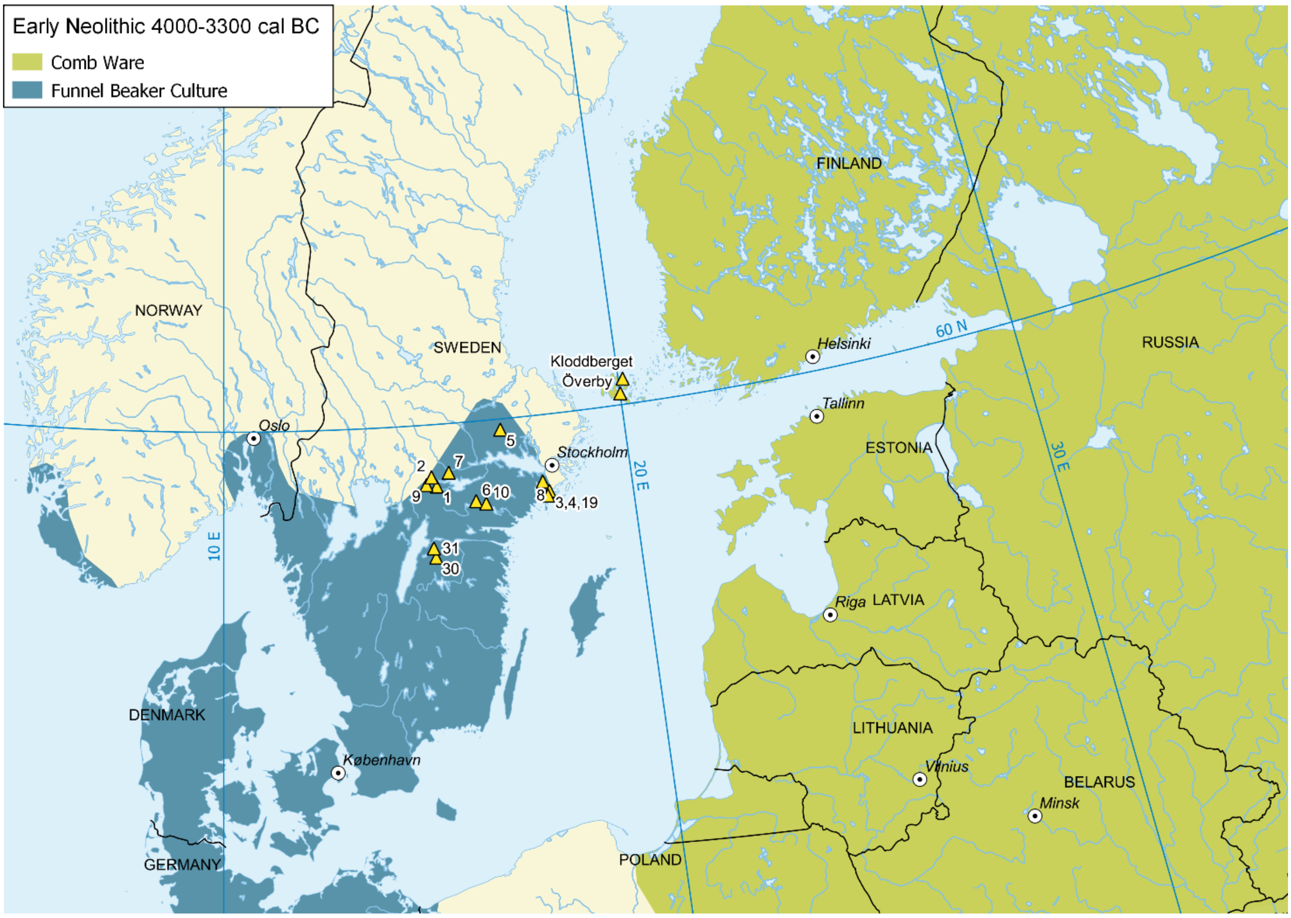

Supplementary figure 1. Early Neolithic period. Distribution of Comb Ware and Funnel Beaker Culture in northern Europe during the Early Neolithic period, c. 4000–3300 cal BC[25,26]. Find locations with numbers demarcate sites where cereal grains have been found and later AMS radiocarbon dated. Figure was created by SV using QGIS 3.4. (https://www.qgis.org/) and Natural Earth data (https://www.naturalearthdata.com/).

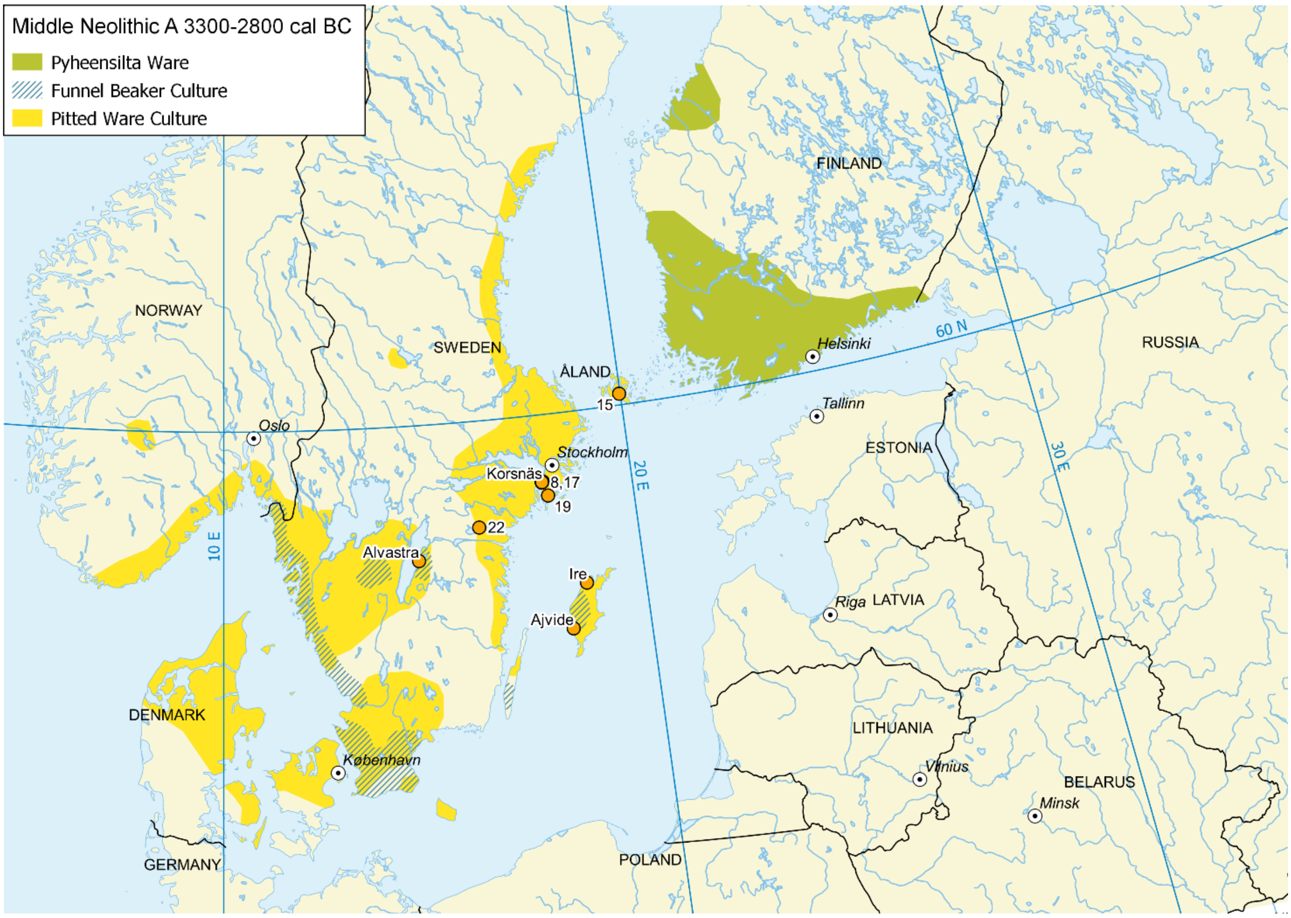

Supplementary figure 2. Middle Neolithic A period. Distribution of Pyheensilta Ware, Funnel Beaker Culture in Sweden, and Pitted Ware Culture in northern Europe during the Middle Neolithic A period, c. 3300–2800 cal BC[27–29]. Find locations with numbers demarcate sites where cereal grains have been found and later AMS radiocarbon dated. Figure was created by SV using QGIS 3.4. (https://www.qgis.org/) and Natural Earth data (https://www.naturalearthdata.com/).

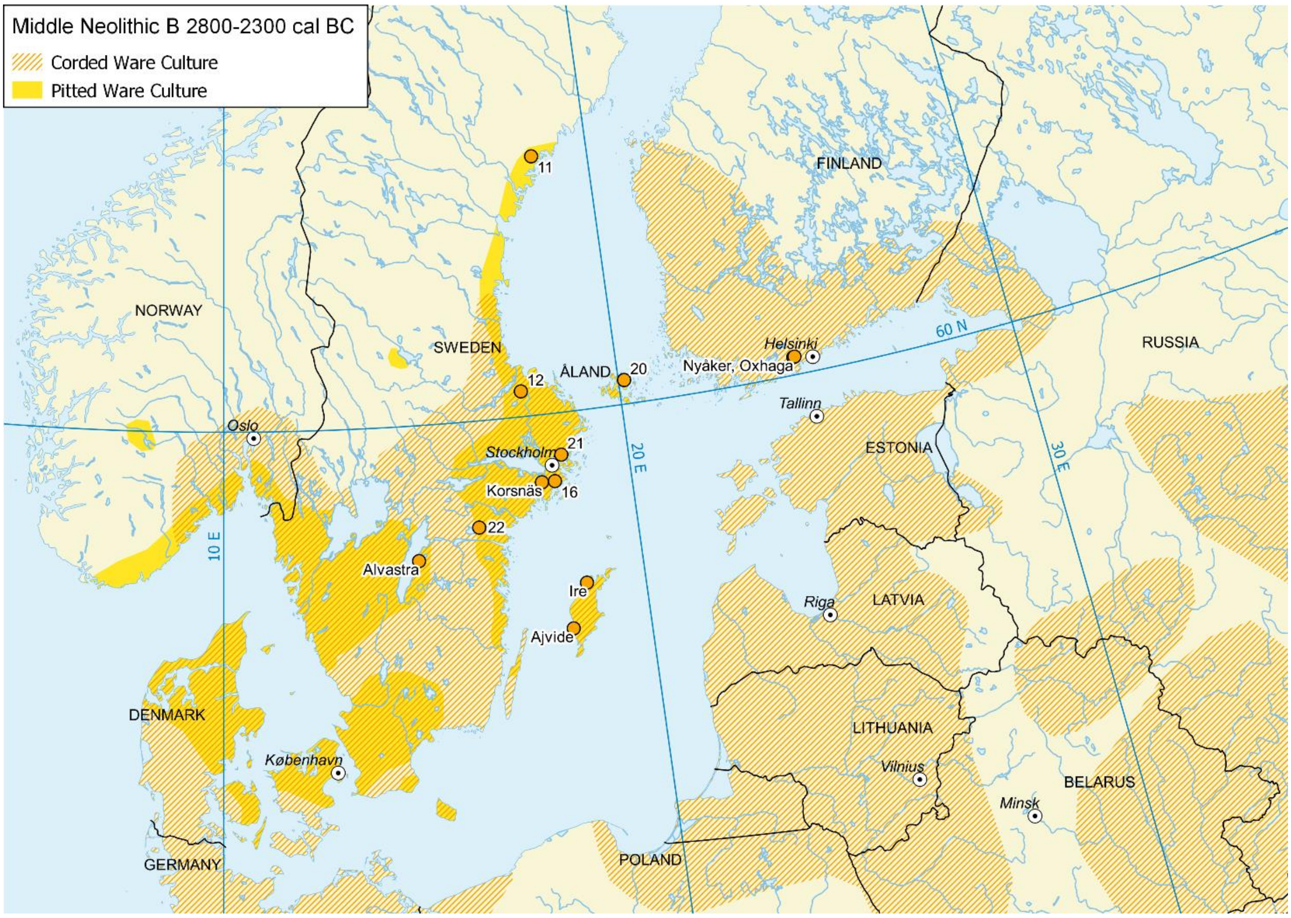

Supplementary figure 3. Middle Neolithic B period. Distribution of Corded Ware Culture and Pitted Ware Culture in northern Europe during the Middle Neolithic B period, c. 2800–2300 cal BC[27,30]. Find locations with numbers demarcate sites where cereal grains have been found and later AMS radiocarbon dated. Figure was created by SV using QGIS 3.4. (https://www.qgis.org/) and Natural Earth data (https://www.naturalearthdata.com/).

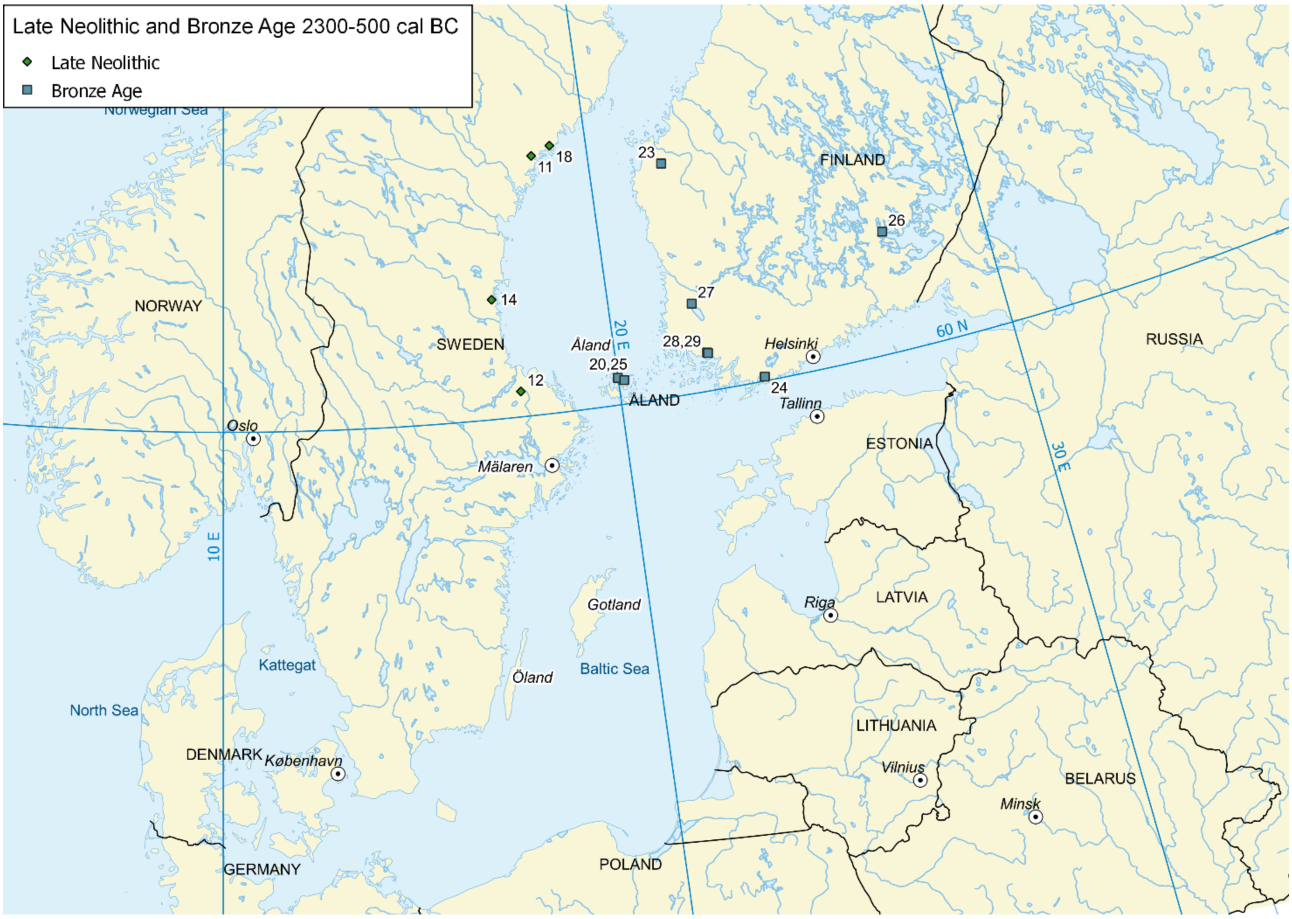

Supplementary figure 4. Late Neolithic and Bronze Age periods. Find locations with numbers demarcate sites where cereal grains have been found and later AMS radiocarbon dated to the Late Neolithic and the Bronze Age periods. Figure was created by SV using QGIS 3.4. (https://www.qgis.org/) and Natural Earth data (https://www.naturalearthdata.com/).

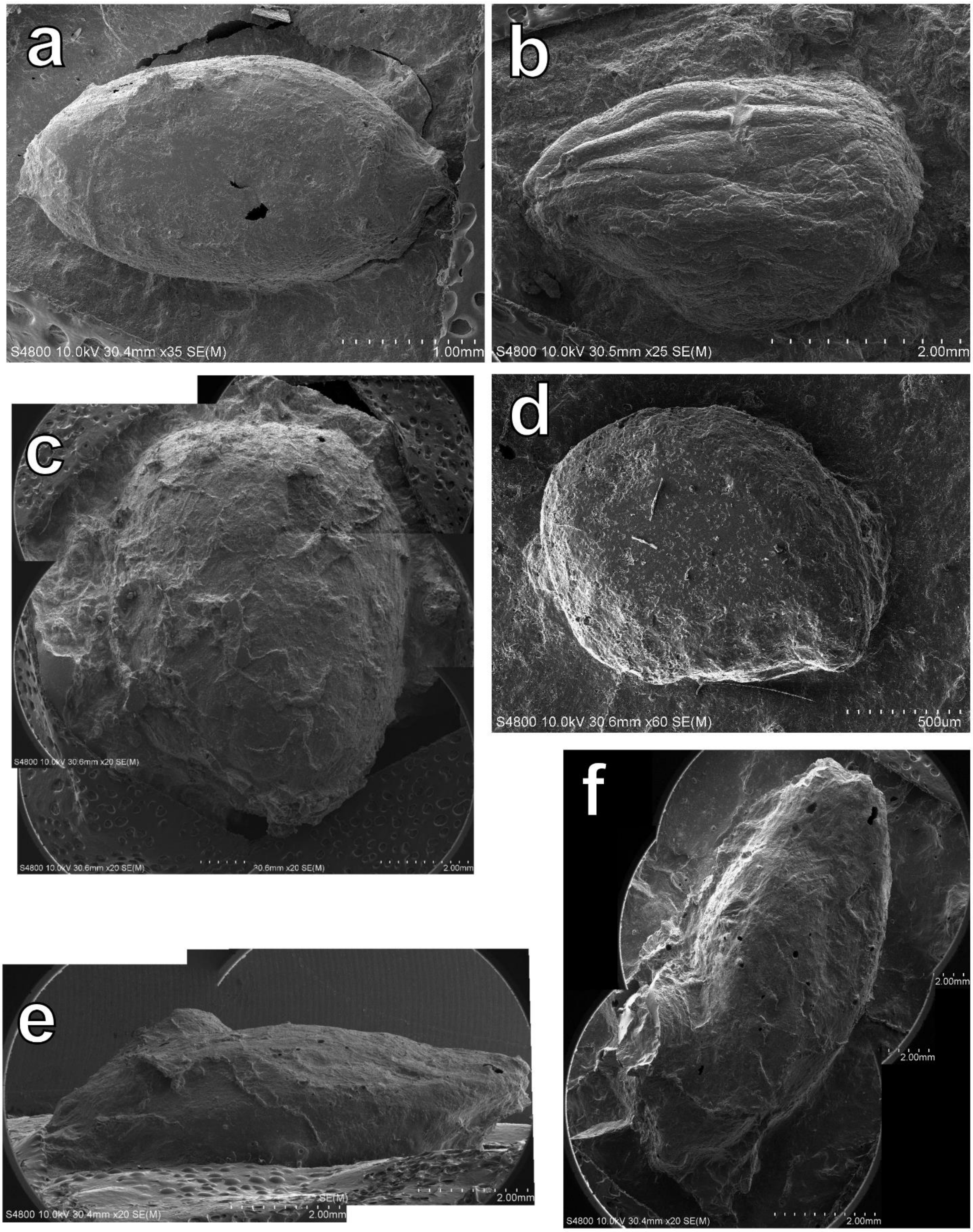

Supplementary Figure 5. SEM-images of silicone casts: a) *Melica nutans,* 3.3×1.7 mm (Tengo Nyåker); b) *Juniperus communis* 4.5×2.5 mm (Tengo Nyåker); c) cf. *Prunus padus* 8.9×7.3 mm (Tengo Nyåker); d) *Fragaria vesca* 1.4×1.6 mm (Kauhala Oxhaga); e–f) lateral and dorsal *Hordeum vulgare* 8.9×3.9 mm (Ristimäki). Images: Marianna Kemell.

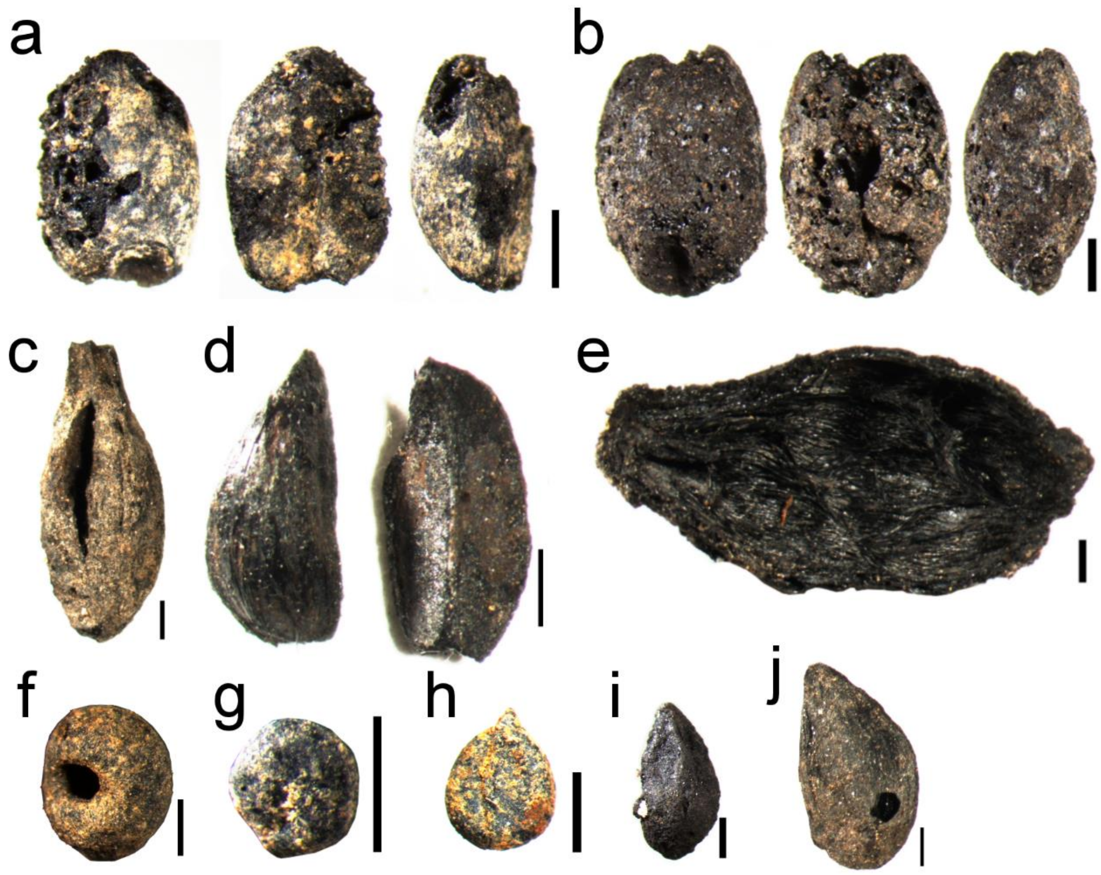

Supplementary Figure 6. Selected charred remains from Åland: a) *Triticum aestivum* s.l. (Svinvallen, dated Ua-53759); b) *Hordeum vulgare* var. *nudum* (Jettböle I, dated Ua-53765); c) *Ranunculus ficaria* (Glamilders); d) *Rosa* sp. (Glamilders); e) *Rosa* sp. receptacle (Glamilders); f) *Galium spurium* (Svinvallen); g) *Chenopodium* sp. (Svinvallen); h) *Persicaria* sp. (Glamilders); i) *Juniperus communis* (Överby); and, j) *Malus sylvestris* (Glamilders). Scale bars 1 mm. Images: Santeri Vanhanen.

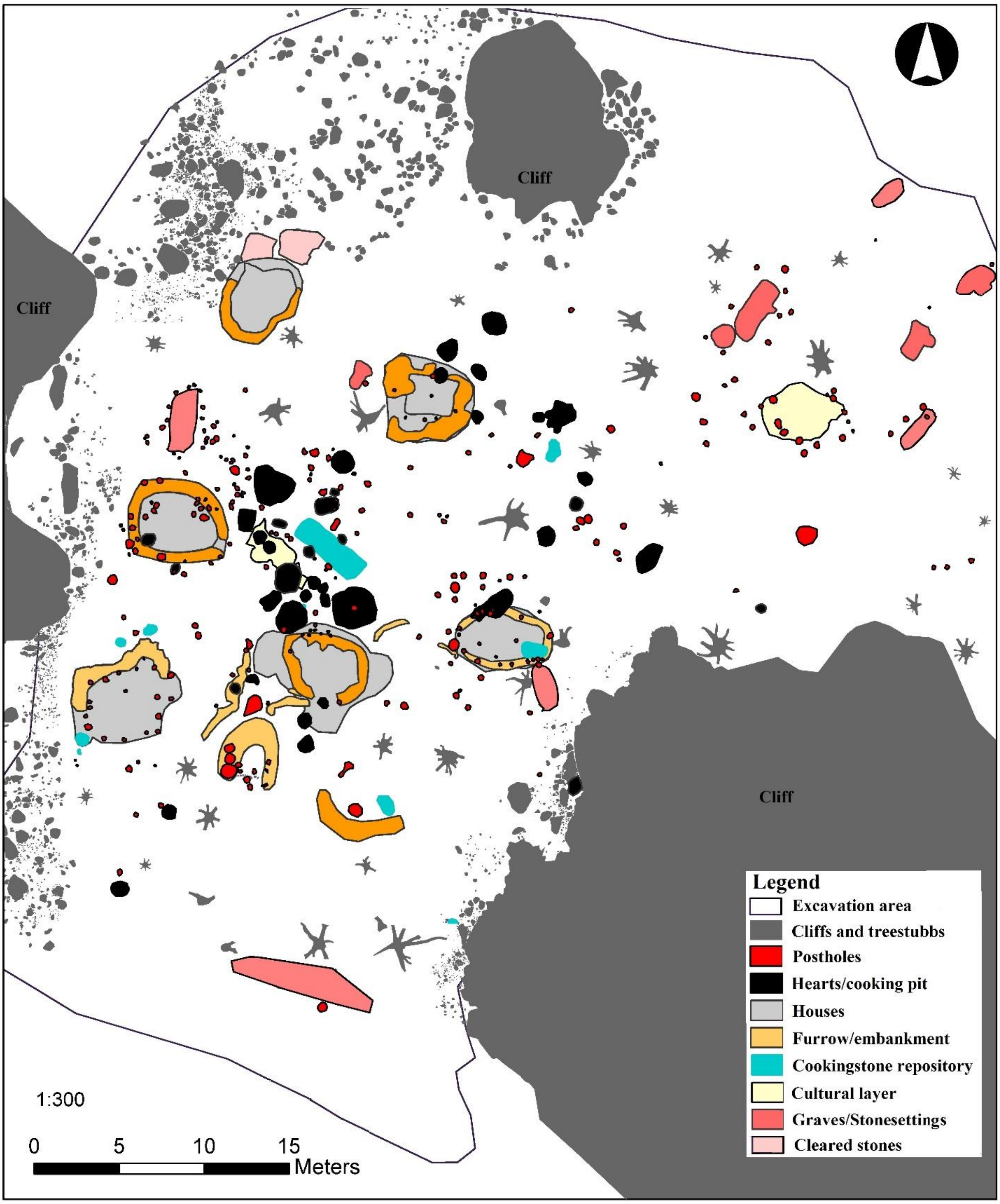

Supplementary figure 7. Excavation plan from Tråsättra. Image: Niclas Björck.

Supplementary tables

Supplementary table 1. Radiocarbon dates from studied sites.

Dates are published in the current article and have been obtained from the literature. Excavation reports were accessed from the archives of Ålands Museum in Mariehamn and the Finnish National Board of Antiquities in Helsinki as well as extant literature. Radiocarbon dates were calibrated with OxCal 4.2. with IntCal 13 curve[31]. Dates marked with asterisk (*) published in the current article.

Site	Lab number	BP	Dated material	Calibrated age (2 σ)
Kloddberget[1]	Ua-35047	6125±40	Charcoal	5210–4958 cal BC
	Ua-35046	5580±40	Charcoal	4488–4347 cal BC
Överby[3]	Ua-19514	4925±50	Charcoal	3905–3637 cal BC
Jettböle I[5–7,32,33]	Ua-11465	4375±70	Alces alces	3333–2887 cal BC
	Ua-10687	4275±65	Alces alces	3090–2640 cal BC
	Ua-10688	3825±70	Alces alces	2471–2044 cal BC
	Ua-15872	4479±60	Ceramic crust	3360–2938 cal BC
	Ua-15871	4425±65	Ceramic crust	3338–2913 cal BC
	Ua-15873	4245±60	Ceramic crust	3012–2634 cal BC
	Ua-15881	4375±75	Corylus avellana	3335–2885 cal BC
	Ua-15880	4340±70	Corylus avellana	3331–2777 cal BC
	Ua-15879	4305±75	Corylus avellana	3322–2669 cal BC
	CAMS 6264	4520±60	Homo sapiens	3488–3023 cal BC
	CAMS 6262	4430±60	Homo sapiens	3338–2917 cal BC
	CAMS 6263	4320±60	Homo sapiens	3265–2708 cal BC
	Ua-15874	4035±75	Homo sapiens	2872–2349 cal BC
	Ua-21512	3285±45	Homo sapiens	1682–1451 cal BC
	Ua-53765*	4424±31	Hordeum vulgare var. nudum	3324–2923 cal BC
Jettböle II[5,6,10]	Ua-15882	4125±80	Ceramic crust	2890–2491 cal BC
	Ua-19513	3970±45	Corylus avellana	2617–2309 cal BC
	Ua-19512	3970±65	Ceramic crust	2836–2234 cal BC
	Ua-19511	3925±50	Ceramic crust	2570–2233 cal BC
	Ua-10686	3400±60	Bos taurus	1881–1534 cal BC
	Ua-11461	925±80	Sus scrofa	985–1261 cal AD
Glamilders[11,34]	Ua-53760*	4167±31	Hordeum vulgare	2881–2634 cal BC
	Ua-53761*	4102±31	Hordeum vulgare var. nudum	2864–2503 cal BC
	Ua-53762*	4139±31	Hordeum vulgare	2875–2620 cal BC
	Ua-35045	4145±35	Hordeum vulgare var. nudum	2877–2620 cal BC
	Ua-23508	4035±40	Corylus avellana	2836–2468 cal BC
	Ua-23510	4110±45	Charcoal	2872–2504 cal BC
	Ua-23509	4080±45	Charcoal	2864–2487 cal BC
	Ua-23507	3950±45	Charcoal	2573–2300 cal BC
Åby[13,14]*	Ua-54407	4400±30	Corylus avellana	3262–2917 cal BC
	Ua-51518	4399±30	Corylus avellana	3261–2917 cal BC
	Ua-51522	4392±36	Hordeum vulgare var. nudum	3264–2909 cal BC
	Ua-51523	4363±34	Hordeum vulgare var. nudum	3089–2903 cal BC
	Ua-54406	4361±29	Corylus avellana	3084–2906 cal BC

	Ua-51526	4355±34	Triticum sp.	3086–2900 cal BC
	Ua-54799	4338±39	Ceramic crust	3085–2890 cal BC
	Ua-51519	4309±33	Corylus avellana	3013–2886 cal BC
	Ua-51525	4292±34	Hordeum vulgare var. nudum	3011–2877 cal BC
	Beta-356260	4280±30	Charcoal, Quercus sp.	3008–2872 cal BC
	Ua-51520	4274±33	Cerealia	3009–2762 cal BC
	Beta-353396	4250±30	Triticum sp.	2916–2714 cal BC
	Ua-51524	4220±34	Hordeum vulgare var. nudum	2906–2679 cal BC
	Ua-54405	4211±29	Corylus avellana	2900–2681 cal BC
	Ua-54798	4193±32	Ceramic crust	2893–2671 cal BC
	Ua-51521	4123±33	Cerealia	2871–2579 cal BC
	Ua-9451	4105±60	Ceramic crust	2877–2494 cal BC
	Beta-353394	3940±30	Cerealia	2565–2309 cal BC
	Ua-9166	3800±85	Ceramic crust	2474–1982 cal BC
	Ua-51706	2517±31	Charcoal, Corylus avellana	794–541 cal BC
Tråsättra[15]*	Ua-55117	3891±32	Hordeum vulgare	2471–2286 cal BC
	Ua-55116	4009±32	Hordeum vulgare	2618–2468 cal BC
	Ua-55113	4113±33	Hordeum vulgare	2866–2576 cal BC
	Ua-55118	4129±33	Hordeum vulgare	2872–2581 cal BC
	Ua-55115	4171±33	Triticum aestivum s.l.	2884–2634 cal BC
	Ua-56215	4110±32	Corylus avellana	2870–2570 cal BC
	Ua-56221	4104±28	Corylus avellana	2870–2570 cal BC
	Ua-55114	4091±32	Corylus avellana	2870–2490 cal BC
	Ua-56222	4085±28	Corylus avellana	2860–2490 cal BC
	Ua-56220	4083±28	Corylus avellana	2860–2490 cal BC
	Ua-52466	4065±31	Corylus avellana	2850–2480 cal BC
	Ua-56223	4065±31	Corylus avellana	2850–2480 cal BC
	Ua-56212	4058±27	Corylus avellana	2840–2480 cal BC
	Ua-56213	4044±28	Corylus avellana	2840–2470 cal BC
	Ua-56214	4043±28	Corylus avellana	2840–2470 cal BC
	Ua-52468	4026±31	Corylus avellana	2630–2470 cal BC
	Ua-56218	4022±28	Corylus avellana	2575–2490 cal BC
	Ua-56224	4022±21	Corylus avellana	2575–2485 cal BC
	Ua-55116	4009±32	Hordeum vulgare	2618–2468 cal BC
	Ua-52465	3997±31	Corylus avellana	2565–2460 cal BC
	Ua-56216	3991±28	Corylus avellana	2580–2460 cal BC
	Ua-52469	3989±31	Corylus avellana	2580–2460 cal BC
	Ua-56219	3947±28	Corylus avellana	2570–2340 cal BC
	Ua-55117	3891±32	Hordeum vulgare	2565–2450 cal BC
	Ua-56225	3885±26	Corylus avellana	2470–2290 cal BC
Svinvallen	Ua-53759*	4082±31	Triticum sp.	2859–2495 cal BC
	Ua-53758*	2652±72	Hordeum vulgare var. nudum	997–551 cal BC
Bäljars 2[20,35]	Poz-66757*	3090±35	Hordeum vulgare	1431–1264 cal BC
	Hela-2221	2240±33	Cerealia	392–204 cal BC
	Hela-2225	2211±32	Corylus avellana	375–198 cal BC

	Poz-66756	1820±30	Triticum dicoccum/spelta	90–321 cal AD
	Hela-2224	617±30	Corylus avellana	1293–1401 cal AD
Härdalen	Ua-53763*	2403±29	Hordeum vulgare var. nudum	731–400 cal BC
	Ua-53764*	2707±29	Hordeum vulgare var. nudum	907–808 cal BC
Ristimäki[24]	Hela-3525	2447±33	Hordeum vulgare	754–410 cal BC
	Poz-66814	2880±30	Ceramic crust	1192–939 cal BC

Supplementary table 2. Cerealia datings.

Dated cereals from the study area are published in this article and have been obtained from the literature. Dates marked with asterisk (*) published in the current article.

Site number	Site	Material dated	Lab code	BP	cal BC (2 sigma)	Median cal BC
	**Early Neolithic 3950–3300 cal BC**					
1	Attersta[36]	Cerealia	Ua-39358	5050±46	3959–3714	3861
3	Lisseläng 2[37]	*Hordeum vulgare* var. *nudum*	Ua-32969	5025±45	3946–3710	3834
30	Hulje[38]	*Hordeum vulgare* var. *nudum*	Ua-31067	4963±86	3961–3635	3762
3	Lisseläng 2[37]	*Triticum dicoccum/spelta*	Ua-32967	4940±40	3795–3646	3714
7	Skogsmossen[39]	*Triticum dicoccum/spelta*	Ua-15200	4880±110	3948–3378	3676
6	Nävertorp[40]	*Triticum* cf. *monoccum*	Ua-28712	4860±75	3894–3380	3652
9	Säby[41]	Cerealia	Ua-28358	4830±45	3704–3521	3597
8	Stensborg[42]	*Triticum dicoccum/spelta*	LuS-9184	4800±50	3693–3381	3572
30	Hulje[38]	*Hordeum vulgare var. vulgare*	Ua-38680	4784±35	3648–3385	3568
3	Lisseläng 2[39]	*Hordeum vulgare* var. *nudum*	Ua-14835	4795±75	3708–3373	3567
2	Hjulberga[39]	*Triticum aestivum* s.l.	Ua-3369	4780±65	3692–3374	3560
9	Säby[41]	Cerealia	Ua-28357	4755±45	3642–3377	3557
7	Skogsmossen[39]	Cerealia	Ua-15198	4775±70	3694–3372	3556
8	Stensborg[42]	*Triticum dicoccum/spelta*	LuS-9570	4760±50	3645–3377	3556
4	Lässmyran 1[37]	*Hordeum vulgare* var. *nudum*	Ua-32972	4735±65	3641–3371	3524
7	Skogsmossen[39]	*Triticum aestivum* s.l.	Ua-15199	4735±75	3646–3367	3521
8	Stensborg[42]	*Triticum dicoccum/spelta*	LuS-9571	4710±75	3641–3360	3497
2	Hjulberga[39]	*Hordeum vulgare* var. *nudum*	Ua-3368	4695±65	3635–3364	3479
7	Skogsmossen[39]	*Triticum aestivum* s.l.	Ua-14834	4680±70	3640–3341	3467
19	Sittesta[43]	*Hordeum vulgare* var. *vulgare*	Poz-18293	4700±40	3632–3370	3464
5	Nyskottet[39]	*Triticum aestivum* s.l.	Ua-17860	4670±95	3648–3105	3452
31	Russingstorp[44]	*Cerealia*	Ua-41713	4646±65	3635–3121	3447
10	Östra Vrå[45]	*Triticum dicoccum/spelta*	Ua-6937	4600±60	3620–3101	3366
	**Middle Neolithic 3300–2300 cal BC**					
17	Kyrktorp[46]	*Triticum* sp.	Ua-1405	4575±105	3632–2944	3289
8	Stensborg[42]	*Triticum dicoccum/spelta*	LuS-8636	4510±50	3364–3029	3211
8	Stensborg[42]	*Triticum dicoccum/spelta*	LuS-8637	4510±50	3364–3029	3211
15	Jettböle I*	*Hordeum vulgare* var. *nudum*	Ua-53765	4424±31	3324–2923	3058
19	Sittesta[43]	*Triticum dicoccum/spelta*	Poz-23716	4390±35	3262–2911	3000
19	Sittesta[43]	Cerealia	Poz-23717	4375±35	3091–2909	2985
22	Åby*	*Hordeum vulgare* var. *nudum*	Ua-51523	4363±34	3089–2903	2976
22	Åby*	*Triticum* sp.	Ua-51526	4355±34	3086–2900	2971
19	Sittesta[43]	Cerealia	Poz-18291	4350±40	3090–2894	2972
22	Åby*	*Hordeum vulgare* var. *nudum*	Ua-51522	4329±36	3079–2889	2949
22	Åby*	*Hordeum vulgare* var. *nudum*	Ua-51525	4292±34	3011–2877	2906
22	Åby*	Cerealia	Ua-51520	4274±33	3009–2762	2897
22	Åby*	*Triticum* sp.	Beta-353396	4250±30	2916–2714	2887

22	Åby*	*Hordeum vulgare* var. *nudum*	Ua-51524	4220±34	2906–2679	2798
21	Tråsättra*	*Triticum aestivum* s.l.	Ua-55115	4171±33	2875–2699	2765
13	Glamilders*	*Hordeum vulgare*	Ua-53760	4167±31	2881–2634	2762
13	Glamilders[34]	*Hordeum vulgare* var. *nudum*	Ua-35045	4145±35	2877–2620	2739
13	Glamilders*	*Hordeum vulgare*	Ua-53762	4139±31	2875–2620	2734
21	Tråsättra*	*Hordeum vulgare*	Ua-55118	4129±33	2860–2632	2723
22	Åby*	Cerealia	Ua-51521	4123±33	2871–2579	2712
21	Tråsättra*	*Hordeum vulgare*	Ua-55113	4113±33	2851–2619	2690
13	Glamilders*	*Hordeum vulgare* var. *nudum*	Ua-53761	4102±31	2864–2503	2667
20	Svinvallen*	*Triticum aestivum* s.l.	Ua-53759	4082±31	2859–2495	2628
16	Jordbromalm[47]	*Triticum dicoccum/spelta*	n/a	4040±40	2840–2469	2563
21	Tråsättra*	*Hordeum vulgare*	Ua-55116	4009±32	2570–2484	2530
11	Bjästamon[48]	Cerealia	Ua-27101	3985±45	2622–2346	2517
22	Åby*	Cerealia	Beta-353394	3940±30	2565–2309	2441
12	Djurstugan[49]	*Triticum aestivum* s.l.	Poz-6249	3910±35	2481–2289	2397
21	Tråsättra*	*Hordeum vulgare*	Ua-55117	3891±32	2471–2286	2383
11	Bjästamon[48]	*Hordeum vulgare*	Ua-27090	3860±45	2465–2205	2337
	**Late Neolithic 2300–1700 cal BC**					
12	Djurstugan[49]	*Hordeum vulgare*	Poz-6251	3825±35	2456–2146	2273
18	Lill-Mossjön[49]	*Hordeum vulgare* var. *nudum*	Ua-25338	3790±55	2456–2039	2228
14	Hedningahällan[50]	Hordeum vulgare var. nudum	Ua-5080	3780±110	2551–1910	2217
11	Bjästamon[48]	*Hordeum vulgare*	Ua-27100	3750±45	2294–2028	2162
	**Bronze Age 1700–500 cal BC**					
28	Niuskala[51]	*Hordeum vulgare* cf. *nudum*	Hela-338	3200±170	1890–1021	1471
24	Bäljars 2*	*Hordeum vulgare*	Poz-66757	3090±35	1431–1264	1346
26	Kitulansuo[52]	*Hordeum vulgare*	Hela-167	3010±80	1431–1016	1243
29	Ristimäki[24]	Ceramic crust	Poz-66814	2880±30	1192–939	1057
23	Alatalo/Palomäki W[53]	*Hordeum vulgare*	Poz-23351	2785±30	1007–845	935
25	Härdalen*	*Hordeum vulgare* var. *nudum*	Ua-53764	2707±29	907–808	856
20	Svinvallen*	*Hordeum vulgare* var. *nudum*	Ua-53758	2652±72	997–551	828
23	Alatalo/Palomäki W[53]	*Hordeum vulgare*	Ua-33250	2590±40	832–553	785
27	Luistari[54]	*Hordeum vulgare*	Hela-208	2560±55	824–516	678
23	Alatalo/Palomäki W[53]	*Avena* sp.	Ua-34506	2455±40	757–413	591
29	Ristimäki*	*Hordeum vulgare*	Hela-3525	2447±33	754–410	566
25	Härdalen*	*Hordeum vulgare* var. *nudum*	Ua-53763	2403±29	731–400	473

Supplementary table 3. Archaeobotanical materials.

Charred plant remains material from the study area dated to the Early Neolithic and Middle Neolithic periods. Skogsmossen[39], Hjulberga[39], Stensborg[42], Alvastra[55] have been published elsewhere, while Glamilders, Åby, and Tråsättra have been published in this study. For Alvastra, charred remains studied by Hans Göransson[55] are included in the table. Cultivated plant remains from Alvastra have not been dated, and the dates from the site were obtained from hazelnuts, charcoal, human bones, and wood[56]. * present in 9 samples.

Cal B.C. (2 sigma) of C14 dated plant remains	3948–3341	3692–3364	3693–3029	3350–2750	2881–2503	3262–2309	2884–2286
Site	Skogsmossen	Hjulberga	Stensborg	Alvastra	Glamilders	Åby	Tråsättra
Archaeological culture	FBC	FBC	FBC	FBC & PWC	PWC	PWC	PWC
Volume of studied soil (litres)	-	-	2.4	-	28	208	138
Number of analysed samples	-	-	3	143	12	113	46
**Charred plant remains**	17	68	7389	9456	241	97	12
Remains per litre	-	-	3078.8	-	8.7	0.5	0.1
**Cultivated plants**							
naked barley (*Hordeum vulgare* var. *nudum*)			140	6884	1	16	
cf. naked barley (*H. vulgare* cf. *nudum*)			59		1		
barley ear fragments (*H. vulgare*)				16			
barley (*H. vulgare*)	13	37			4	21	5
barley lemma/palea (*H. vulgare*)				61			
barley rachis (*H. vulgare*)				287			
cf. barley (cf. *H. vulgare*)					1		
hulled barley (*H. vulgare* var. *vulgare*)						2	
naked wheat (*Triticum aestivum* s.l.)	3	30	255			3	1
cf. naked wheat (cf. *T. aestivum* s.l.)						1	
cf. club wheat (cf. *T. compactum*)				1			
emmer wheat (*T. dicoccum*)	1	1	2430	1256			
emmer wheat glume base (*T. dicoccum*)			133				
emmer wheat spikelet fork (*T. dicoccum*)			153	166			
emmer wheat rachis internode (*T. dicoccum*)			1				
emmer/spelt wheat (*T. dicoccum/spelta*)			291				
cf. spelt wheat (*T.* cf. *spelta*)			106				
wheat (*Triticum* sp.)					1	4	
cereal (Cerealia), including fragments			3813	57	13	28	4
cereal straw (Cerealia)			8				
**Wild gathered plants**							
tuber oat grass basal internode (*Arrhenatherum elatius* var. *bulbosum)*					1		
hazelnut shell fragment (*Corylus avellana*)				22	177	18	9*
floating sweet-grass (*Glyceria fluitans*)				1			
cf. reed sweet-grass (cf. *Glyceria maxima*)				8			
crab apple *(Malus sylvestris)*				44	2		

crab apple chips *(M. sylvestris)*				14			
lesser celandine root tuber (*Ranunculus ficaria)*					2		1
cf. lesser celandine root tuber (cf. *R. ficaria)*					1		
rose (*Rosa* sp.)					22		
raspberry (*Rubus idaeus*)				1			
mistletoe (*Viscum album*)				1			
**Aquatic and wetland plants**							
great fen-sedge (*Cladium mariscus*)				1			
cf. reed canary-grass (cf. *Phalaris arundinaceae*)				1			
branched bur-reed (*Sparganium erectum*)					1		
least bur-reed (*Sparganium natans*)					1		
bur reed (*Sparganium* sp.)					1		
**Arable weeds**							
mugwort (*Artemisia vulgaris*)				459			
black-bindweed (*Fallopia convolvulus*)				78			
fat hen (*Chenopodium album*)				87			
creeping thistle (*Cirsium arvense*)				1			
false cleavers (*Galium spurium*)					2		
cleavers (*Galium aparine*)							1
cf. darnel *(*cf. *Lolium temulentum)*				1			
small water-pepper (*Persicaria minor*)					1		
knotgrass (*Polygonum aviculare*)					1		
ivy-leaved speedwell (*Veronica hederifolia*)				1			
**Other plants**							
cf. Agrostis sp.				1			
Galium sp.					2		
shining crane's-bill (*Geranium lucidum*)				1			
Fabaceae				1			
cf. Fabaceae					1		
field/heath wood-rush (*Luzula campestris/multiflora*)				1			
*Populus/Betula*branch					1		
Trifolium sp.						1	
Vicia sp.						2	
indeterminate				4	4	1	

Supplementary table 4. Kloddberget.

Charred archaeobotanical materials from Kloddberget. Remains are seeds or fruits, unless noted otherwise.

Number (ÅM 737:)	125	126	128	129	131	132	133	135	136	142	145	146	total
Trench	PG10	PG10	PG10	PG10	PG10	PG10	PG10	PG10	PG10	PG18	PG18	PG18	
Square	B	B	B	B	B	B	B	B	B				
Context	7	6	7	7	6	7	6	6	6				
Hearth	A	A	A	A	A	A	A	A	A	B	B	B	
Layer		5			5		4	5	4	4	5	5	
Weight	3164	3343	2388	4445	4704	2342	2498	798	877	874	571	634	26636,7
Volume (litres)	2.6	2.2	2	3.6	3	1.2	2.1	0.6	0.6	0.7	1.3	0.6	20.5
Organic volume (ml)	390	100	300	280	130	60	60	5	5	60	200	80	1670
Charcoal (0–3)	3	3	3	3	3	3	2	1	1	2	3	2	
Insects	0	0	0	0	0	0	0	1	1	0	0	1	
**Charred plant remains**													97
knotgrass *(Polygonum aviculare)*		29	3	17	13								62
juniper (*Juniperus communis*)		4							1		1	3	9
hazel shell fragments (*Corylus avellana*)			2										2
horsetail branch fragment (*Equisetum* sp.)	2												2
bud	1												1
resin/slag	1												1
indet.	3		2		8		3	2	1		1		20
**Other remains**													
large charcoal pieces (1=present)	1	1	1	1	1	1	1			1	1	1	10
branches (1=present)	1	1	1	1	1	1	1			1	1	1	10
red ochre?							1						1

Supplementary table 5. Glamilders.

Charred archaeobotanical materials from Glamilders. Remains are seeds or fruits, unless noted otherwise. Radiocarbon datings from samples: sample 4552, Hordeum vulgare 2881–2634 cal BC (Ua-53760); sample 4555, Hordeum vulgare var. nudum 2864–2503 cal BC (Ua-53761); sample 4557, Hordeum vulgare 2875–2620 cal BC (Ua-53762); sample 4559, Corylus avellana 2836–2468 cal BC (Ua-23508); sample 15, Hordeum vulgare cf. nudum 2877–2620 cal BC (Ua-35045); sample 18, Corylus avellana 2836–2468 cal BC (Ua-23508). P: present.

Sample number (ÅM 726:)	4552	4554	4555	4556	4557	4558	4559	13	14	15	17	18	total
Square	D1	D1	D1	D1	DE1	DE2	E1	E0	E0	E0	D1	E1	
Level	4				3	4-5	3	1A/B	2	3	2B	3B	
Volume (litres)	2.2	1.4	1.1	1.5	3.9	2.7	2.9	2.0	2.0	2.0	2.0	4.0	27.7
Organic volume (ml)	20	10	10	10	20	20	10						
Charcoal (0–3)	2	1	1	1	2	2	2						
**Charred plant remains**	22	12	60	7	99	45	3	0	2	2	4	6	262
**Cultivated plants**	2		2		7	5			1	1	1	2	21
naked barley *(Hordeum vulgare var. nudum)*			1										1
cf. naked barley *(H. vulgare cf. nudum)*										1			1
barley *(H. vulgare)*	1					1							2
cf. barley (cf. *H. vulgare*)									1				1
barley frag (*H. vulgare*)					2								2
wheat (*Triticum* sp.)					1								1
cereal (Cerealia)	1					1							2
cereal frag (Cerealia)			1		4	3					1	2	11
**Wild gathered plants**													
hazelnut frag (*Corylus avellana*)	17	11	55	6	54	34		p	p	p	p	p	177
rose (*Rosa* sp.)					16						1		17
cf. sherard's downy-rose *(Rosa* cf. *sherardii)*												1	1
rosa hip (*Rosa* sp. hip)					2								2
rose calyx? (*Rosa* sp.)					1								1
rose thorn (*Rosa* sp.)					1								1
crab apple (*Malus sylvestris)*					2								2
lesser celandine root tuber (*Ranunculus ficaria)*					1						1		2
cf. lesser celandine root tuber (cf. *R. ficaria*)					1								1

tuber oat grass basal internode (*Arrhenatherum elatius* var. *bulbosum*)												1	1
**Aquatic plants**													
branched bur-reed (*Sparganium erectum*)					1								1
least bur-reed (*Sparganium natans*)					1								1
bur-reed (*Sparganium* sp.)							1						1
**Arable weeds (P. minor and P. aviculare also shore plants)**													
small water-pepper (*Persicaria minor*)	1												1
knotgrass (*Polygonum aviculare*)					1								1
false cleavers (*Galium spurium*)					1		1						2
**Other plants**													
Galium sp.		1	1										2
cf. Fabaceae						1							1
*Populus/Betula*branch							1						1
indeterminable				1	3								4
**Other finds (1=present)**													
Bones	1	1	1	1	1								5
Amber					1								1
Ceramics		1	1										2
Charred "branches"							1						1

Supplementary table 6. Svinvallen.

Charred archaeobotanical materials from Svinvallen. Remains are seeds or fruits, unless noted otherwise. Radiocarbon datings from samples: sample JP1 Hordeum vulgare var. nudum 997–551 cal BC (Ua-53758); JP2 Triticum aestivum s.l. 2859–2495 cal BC (Ua-53759).

Sample number	JP1	JP2	JP3	JP4	JP5	JP6	JP7	JP8	JP9	total
Point number	547	548	549	550	551	552	553	554	555	
Context	E-Profile A1, E-W trench, bottom	E-Profile, SW-trench, structure 1, middle of the depression	N-Profile	Profile, 2nd square from E		E-profile	W-Profile	W-Profile	W-Profile	
Volume (L)	3.6	4.3	4.1	4.6	3.9	5.4	4.8	4.9	4.8	40.4
Notes (1=present)										
recent roots	1	1	1	1		1	1	1	1	
branches (charred)	1	1	1			1	1	1		
wood partly unburned				1						
Org. volume (ml)	150	190	190	250	180	190	190	270	250	1860
Charcoal (0–3)	2	2	2	2	2	2	2	2	2	
Insects (1=present)	0	1	0	1	1	1	1	0	1	
**Charred plant remains**										350
naked barley (*Hordeum vulgare* var. *nudum)*	1									1
naked wheat (*Triticum aestivum* s.l.)		1								1
cereal (Cerealia)			1				1			2
cf. cereal frag (cf. Cerealia)	3		2					1		6
hazelnut shell frag (*Corylus avellana*)	126	75	9		3	7	12	74	16	322
grass (Poaceae)	1									1
false cleavers (*Galium spurium*)		1								1
Chenopodium sp.		1								1
rose (*Rosa* sp.)			1							1
Fabaceae			1							1
cone scale					2		1			3
cf. *Vaccinium* sp. leaf						1				1
tuber or similar								4	2	6
bud				1						1
indeterminable	1	1								2
**Archaeological finds (1=present)**										
stone flakes	1	1	1	1	1	1	1	1	1	9

ceramics	1	1	1	1	1	1	1	1	1	9
bones	1	1	1	1	1	1	1	1	1	9
slag		1								1

Supplementary table 7. Härdalen.

Charred archaeobotanical materials from Härdalen. Remains are seeds or fruits, unless noted otherwise. Radiocarbon dating from sample 642 JP2, Hordeum vulgare var. nudum 731–400 cal BC (Ua-53763).

Sample number	642 JP 2	642 JP 3	642 JP 4	649: 302	649: 303	649: 304	649: 306	649: 307	649: 308	649: 312	649: 313	649: 314	total
Context	46	46, hearth	46, hearth										
Section	E	W	W	E	E	E	E	E	E	E	W	W	
Level	3	4 to 5	4 to 5	8	8	8	9	9	9	10	8	9	
Organic volume (ml)	20	40	30	10	20	5	<5	10	10	5	5	10	165
Charcoal (0–3)	2	2	2	2	2	1	1	2	2	1	2	2	21
Charred plant remains													
naked barley (*Hordeum vulgare* var. *nudum)*	1												1
barley frag (*H. vulgare)*			1										1
cereal (Cerealia)			1	1									2
grey club-rush (*Schoenoplectus tabernaemontani*)			1										1
hazelnut shell frag (*Corylus avellana*)				12	10	6	1	3	8	1			41
raspberry (*Rubus idaeus*)	1												1
false cleavers (*Galium spurium*)	1												1
Alchemilla sp.				1		1							2
Rosa sp.								3					3
horsetail branch (*Equisetum* sp.)		1											1
marsh woundwort (*Stachys palustris*)											1		1
Bud?		1											1
Branches (1=present)	1	1				1							3
Ceramics (1=present)	1												1
Bones (1=present)				1	1	1						1	4
Fish bones (1=present)							1	1	1	1			4

Supplementary table 8. Tråsättra, soil samples flotated with 0.5 mm mesh.

X-signs signify the following: x=1–5 finds, xx=5–20 finds, xxx=20-100 finds, and xxxx=>100 finds. Remains are seeds or fruits, unless noted otherwise.

Approx. soil sample volume (litres)	Sample number	Context		hazelnut shell frag (*Corylus avellana*)	barley (*Hordeum vulgare*)	cleavers (*Galium aparine*)	naked wheat (*Triticum aestivum* s.l.)	cereal (Cerealia)	lesser celandine root tuber (*Ranunculus ficaria*)	Charcoal	Bone fragments	Fish vertebra	Ceramics
3		A773								xx	xx		
3		G2650								x	x		x
3		A5250		x						xxxx	xx		
3		A5221								xxxx			
3		A6789		x						xxx	xx	x	
3		A8258	Upper level							xxx	xx		
3		A8258	Middle level							xxx	x		
3		A8258	Lower level							xxxx	xx		
3		A8258								xxx	xxx		x
3		A9116		x						xxx	x	x	
3	PM100	A3252								x	x		x
3	PM5047	A3403								xxx	xx		
3	PM5751	A5262								xxx	xx	x	
3	PM5752	A5254								xxx			x
3	PM5754 (?)	A5243								x	x		x
3	PM5754	A5269								xxxx	xx		
3	PM5946	A2490								xxx			
3	PM5947	A2490								xxx			
3	PM5948	A2660								xx	xx		
3	PM5949	A2660								xx	xxx		
3	PM5950	A2660								xx	xx		
3	PM5951	A2660								x	xx		
3	PM6315	A2465								xxx	x		x
3	PM6683	A5221								xxx	xxx		
3	PM6828	A2465								x	x		x

3	PM7495	A2425								xxx			
3	PM7496	A2425			1					xxx	x	x	
3	PM7497	A2526								xx	x		
3	PM7498	A2425		x						xxx	x		
3	PM7890			x						xx	x		
3	PM7990	A1109								x			
3	PM8249	A2425								xx	x		
3	PM8250	A2425		x						xx	x		
3	PM8262	A2425				1				xxx		x	
3	PM8251	A2425								x			
3	PM8254	A2426		x			1			xxxx			
3	PM8255	A2425								xx	x		
3	PM8283	A2425								xxx	xx		x
3	PM8480	A3387								xxx	xx		
3	PM8664	A8457								xxx	xx		
3	PM8865	A8457			1			1		xxx	xx		
3	PM9529	G8344		x						xxx	xx		
3	PM9645	A2425								xxxx			
3	PM10419	A9896		x						xxxx	xx		
3	PM10462	A7753							1	xxx	xx	x	
3	PM11165	A?			3			3		xxxx			
138					5	1	1	4	1				

Supplementary table 9. Tråsättra, soil samples flotated with 2 mm mesh.

X-signs signify the following: x=1–5 finds, xx=5–20 finds, xxx=20-100 finds, and xxxx=>100 finds. Remains are seeds or fruits, unless noted otherwise.

Context number	Context detail	Context type	Sampling area	Soil volume (litres)	naked wheat (*Triticum aestivum* s.l.)	cereal (Cerealia)	barley (*Hordeum vulgare*)	Hazelnut shell frag (*Corylus avellana*)	Fish vertebra	Charcoal	Bone fragment
A5221		Hearth/Cooking pit	Upper level/West	40	2			xxxx	xxxx	xxxx	xxxx
A5221		Hearth/Cooking pit	Upper level/East	35	2	1		xx	x	xxxx	
A5221		Hearth/Cooking pit	Lower level/West	15				x	x	xxx	xx
A5221		Hearth/Cooking pit	Lower level/East	30	2	2		x	x	xxxx	xxx
A8258		Hearth/Cooking pit		40	2	2		x	x	xxxx	xx
A10159	G237	Post hole		25			1	1		xxxx	
A2465	G6218			20						xxxx	
A1817				20						xxx	x
	G4749			?		1		x	xx	xxx	xxx
total				>225	8	6	1				

Supplementary table 10. Åby soil samples.

Remains are seeds or fruits, unless noted otherwise.

Sample number	Context number	naked barley (Hordeum vulgare var. nudum)	hulled barley (H. vulgare var. vulgare)	barley (H. vulgare)	naked wheat (*Triticum aestivum* s.l.)	wheat (*Triticum* sp.)	cf. naked wheat (*Triticum aestivum* s.l.)	cereal (Cerealia)	cereal frag (Cerealia)	hazelnut shell frag (*Corylus avellana*)	*Trifolium* sp.	*Vicia* sp.	indeterminable
2690	2473									1			
4994	4976			1									
5341	5327								1				
5804	4686	1	1	4					2		1		
6009	5858									1			
7623	6487			1									
8166	5829									1		1	
9183	7229				1				1				
9329	8439		1										
9430	9036	1		1									
9432	9415			1									
9434	8736			1									
9905	8772	1											
10820	7182	1			1				2	1			
10795	8299	1							1				
10797	8299	1		1					2	1			
10834	10384	1		2					2				
10843	7428			2	1				1				
10847	9515									3			
10853	10384	8		6		2			12				
11288	9515	1							3				
11496	7671			1									
11625	11541									1			
12218	9129									1			
12453	12257					1				5			
12517	12491												1
12522	7061									1			
12643	9065						1						

12676	11070									1			
13055	11098									1			
15_4026	A15							1					
17_4350	L17					1						1	
	total	16	2	21	3	4	1	1	27	18	1	2	1

Supplementary table 11. Casts from Kirkkonummi.

Material derives mainly from the sites Tengo Nyåker and Kauhala Oxhaga.

NM-number	Number	Identification
5944:48	1	twig
6139:6	2	twig
6139:6	3	twig
6139:6	4	twig
7852:6	5	wild strawberry (*Fragaria vesca*)
7734:6	6	indet
7868:6	7	indet
7734:11	8	twig
7734:11	9	twig
7734:11	11	twig
7734:11	12	indet
7734:11	13	indet
9107:8	14	indet
9107:8	15	indet
9107:8	16	indet
9107:8	17	indet
9107:15	18	indet, hair
9107:15	19	twig
8709:1	20	indet, hair
6754:3	21	indet
6754:3	22	indet
6754:3	23	twig
6754:3	24	indet
6754:3	25	indet
8709:3	26	twig
8709:3	27	indet
8709:3	28	indet
8709:3	29	indet
8709:3	30	twig?
8709:3	31	indet
8709:3	32	twig?
8709:3	33	twig
8709:3	34	indet
8709:3	35	indet
8709:3	36	twig
8709:3	37	indet
8709:6	38	twig, hair
8709:6	39	twig, hair
8709:6	40	indet
8709:6	41	twig

8709:9	42	twig
8709:9	43	indet
8709:9	44	twig
8709:9	45	twigs
8709:9	46	indet
8709:9	47	indet
8709:9	48	twig
8709:9	49	twigs
8709:9	50	twigs, hair
8709:9	51	indet
8709:11	52	indet
8709:11	53	twig
8709:11	54	twig
8709:11	55	twig
8709:13	56	twig, indet
8709:13	57	indet
8709:13	58	indet
8709:13	59	twig
8709:13	60	indet
8709:17	61	twig
8709:17	62	indet
8709:17	63	indet
8709:17	64	indet
8709:17	65	twig
8709:17	66	indet
8709:17	67	twig
8709:17	68	indet
8709:17	69	indet
8709:17	70	indet
8709:17	71	indet
8709:20	72	twig
8709:22	73	twig
8709:22	74	indet
8709:22	75	twig
8709:22	76	twig
8709:25	77	indet
8709:25	78	twig, indet
8709:25	79	crack in the vessel surface
8709:25	80	indet
8709:25	81	indet
8709:25	82	twig
8709:25	83	indet
8709:25	84	indet
8709:25	85	indet
8709:25	86	indet

8709:25	87	bud?
8709:25	88	indet
8709:25	89	indet
8709:25	90	indet
8709:25	91	indet
8709:25	92	indet
8709:25	93	twig
8709:25	94	twig, hair
8709:25	95	indet
8709:25	96	indet
8709:25	97	twig, hair
8709:25	98	indet
8709:25	99	indet
8709:25	100	indet
8709:25	101	hair
8709:25	102	twig
8709:25	103	indet
8709:25	104	indet
8709:25	105	hair
8709:29	106	*cf.* Bird cherry (cf. *Prunus padus*)
8709:29	107	twig
8709:29	108	indet
8709:29	109	twig
8709:29	110	indet
8709:29	111	indet
8709:29	112	twig
8709:29	113	twig
8709:29	114	indet
8709:29	115	twig
8709:29	116	twig
8709:29	117	twig
8709:29	118	twig
8709:29	119	twig
8709:29	120	twig
8709:29	121	indet
8709:29	122	indet
8709:35	123	indet
8709:35	124	indet
8709:35	125	twig
8709:42	126	indet
8709:42	127	bud?
8709:42	128	bud?
8709:45	129	indet
8709:25	130	twig
8709:30	131	twig

8709:29	132	mountain melick seed (*Melica nutans*)
8709:25	133	juniper seed (*Juniperus communis*)
8709:25	134	indet
8709:17	135	bud?
21501:3	136	indet
21501:10	137	indet
21501:18	138	indet
21501:25	139	indet
21501:27	140	indet
21501:77	141	indet
21501:77	142	twig
21501:82	143	twig
21501:93	144	twig
21501:93	145	twig
21501:112	146	indet
21501:143	147	twig
21501:143	148	twig
21501:143	149	wood
21501:154	150	indet

References

1. Gustavsson, R. *Gränsbestämning av Sa 2.21: En äldre kamkeramisk boplats vid Kloddberget*. (2007).

2. Engelmark, R. *Miljöarkeologiska analyser från boplats Sa 2.21, Bertby, Saltviks kommun, Åland. Miljöarkeologiska laboratoriet. Rapport nr. 2008-010*. (2008).

3. Gustavsson, R. *Rapport över fortsatt undersökning av härd på stenåldersboplats Jo. 37.11, Överby, Jomala.* (2002).

4. Storå, J. & Stenbäck, N. *Provundersökningar för gränsbestämning av stenåldersboplats Jomala 14.1 Jettböle 1999-2000*. (2002).

5. Storå, J. Sealing and animal husbandry in the Ålandic Middle and Late Neolithic. *Fennoscandia Archaeol.* **16**, 57–81 (2000).

6. Stenbäck, N. Människorna vid havet. Platser och keramik på ålandsöarna perioden 3500-2000 f.Kr. Stockholm Studies in Archaeology 28. (University of Stockholm, 2003).

7. Stenbäck, N. & Storå, J. Nya undersökningar vid Jettböleboplatsen på Åland. *Muinaistutkija* 33–39 (2000).

8. *Stenåldersboplatsen å Jettböle kapellansbol (invid Grönbacka torpen) i Jomala socken på Åland. Find catalogue.* (1905).

9. Mannermaa, K. Bird bones from Jettböle I, a site in the Neolithic Åland archipelago in the northern Baltic. *Acta Zool. Cracoviensia* **45**, 85–98 (2002).

10. Storå, J., Stenbäck, N. & Darmark, K. *Provundersökning av stenåldersboplats Jomala 14.1 Jettböle 2000. Jettböle II*. (2002).

11. Vaara, R. *Undersökning vid Sa 20.8 Glamilders Långbergsöda 2004*. (2004).

12. Engelmark, R., Linderholm, J. & Sjöström, S. *Miljöarkeologiska analyser av jordprov från arkeologisk undersökning av fornlämning; Sa 20.8 Gladmilders, Åland. Miljöarkeologiska laboratoriet rapport nr. 2004-037*. (2004).

13. Andersson, H. *Stenålder i Åby - bland gravar och gropkeramik. RAÄ 36:1, Kvillinge socken, Norrköpings kommun, Östergötland. Rapporter från Arkeologikonsult 2013:2662.* (2013).

14. Runeson, H. & Kihlstedt, B. *Åby: En klassisk gropkeramisk lokal i det inre av Bråviken. Arkeologisk undersökning. Fornlämning Kvillinge 36:1 Häradsmarken 1:23 Kvillinge socken Norrköpings kommun Östergotlands län Östergotland. Rapport 2017:28. Västerås.* (2018).

15. Björck, N. *Tråsättra - Aspekter på de neolitiska säljägarnas vardag och symbolik. Arkeologisk undersökning. Stockholms län, Uppland, Österåkers kommun, Österåker socken, Tråsättra 1:14, Österåker 553.*

16. Äyräpää, A. *Kaivaus Tengon talon Nyåkerin kivikautisella asuinpaikalla Kirkkonummen pitäjän Lappbölen kylässä*. (1926).

17. Edgren, T. On the economy and subsistence of the Battle-Axe Culture in Finland. in *Fenno-ugri et slavi 1983* (ed. Edgren, T.) 9–15 (1984).

18. Cramp, L. J. E. *et al.* Neolithic dairy farming at the extreme of agriculture in northern Europe. *Proc. Biol. Sci.* **281**, 20140819 (2014).

19. Europaeus, A. Fornfynd från Kyrkslätt och Esbo socknar. *Suom. Muinaismuistoyhdistyksen Aikakausk.* **XXXII**, 1–208 (1922).

20. Vanhanen, S. & Koivisto, S. Pre-Roman Iron Age settlement continuity and cereal cultivation in coastal Finland as shown by multiproxy evidence at Bäljars 2 site in SW Finland. *J. Archaeol. Sci. Reports* **1**, 38–52 (2015).

21. Nunez, M. *Sa 21.11 Nääs, Härdalen. Utgrävning av skärvstensröset anl. 46 samt provundersökning av boplatsområdet från sten/bronsåldern*. (1990).

22. Mannermaa, K. & Lõugas, L. Birds in the subsistence and cultures in four major Baltic Sea Islands during the Neolithic. in *Feathers, Grit and Symbolism. Birds and Humans in the Ancient Old and New Worlds. Documenta Archaeobiologiae 3.* (eds. Grupe, G. & Peters, J.) 179–198 (2005).

23. Juhola, T., Klemola, N. & Etu-Sihvola, H. *Kaarinan Ravattulan Ristimäen kivikautisen asuinpaikka-alueen tutkimukset 2013.* (2014).

24. Juhola, T., Etu-Sihvola, H., Näreoja, T. & Ruohonen, J. Starch analysis reveals starchy foods and food processing from Finnish archaeological artifacts. *Fennoscandia Archaeol.* 79–100 (2014).

25. Gron, K. J. & Sørensen, L. Cultural and economic negotiation: A new perspective on the Neolithisation of southern Scandinavia. *Antiquity* **92**, 958–974 (2018).

26. Piezonka, H. *Jäger, Fischer, Töpfer: Wildbeuterguppen mit früher Keramik in Nordosteuropa im 6. und 5. Jahrtausend v. Chr.* (Deutsches Archäologisches Institut, Eurasien-Abteilung, 2015).

27. Sørensen, L. From hunter to farmer in northern Europe. Migration and adaptation during the Neolithic and Bronze Age. *Acta Archaeol.* **I**–**II**, (2014).

28. Pesonen, P. Suomen esihistoriallinen keramiikka. (1999). Available at: http://www.helsinki.fi/hum/arla/keram/.

29. Sjögren, K.-G. Megalithic Landscapes in Sweden. in *Sozialarchäologische Perspektiven: Gesellschaftlicher Wandel 5000-1500 v. Chr. zwischen Atlantik und Kaukasus. Internationale Tagung 15.–18. Oktober 2007* 125–139 (Verlag Philipp von Zabern, 2011).

30. Müller, J. *et al.* A revision of corded ware settlement pattern-new results from the Central European low mountain range. *Proc. Prehist. Soc.* **75**, 125–142 (2009).

31. Bronk Ramsey, C. Bayesian analysis of radiocarbon dates. *Radiocarbon* **51**, 337–360 (2009).

32. Lidén, K., Núñez, M. & Nelson, E. D. Diet and nutritional stress in a subneolithic population from the Åland Islands - an analysis of stable carbon isotopes and pathological traits. in *Proceedings from the 6th Nordic Conference on the Application of Scientific Methods in Archaeology Esbjerg 1993* (eds. Mejdahl, V. & Siemen, P.) 23–37 (Esbjerg Museum, 1996).

33. Götherström, A., Stenbäck, N. & Storå, J. The Jettböle middle Neolithic site on the Åland Islands - human remains, ancient DNA and pottery. *Eur. J. Archaeol.* **5**, 42–69 (2002).

34. Lucenius, J. Keramiken i rummet. En analys av gropkeramik och fyndkontexter på Glamilders Österlen på Åland ca 2800-2300 BC. (University of Helsinki, 2008).

35. Alenius, T., Haggrén, G., Koivisto, S., Vanhanen, S. & Sugita, S. Landscape dynamics in southern Finland during the Iron Age and the Early Modern Era — Pollen-based landscape reconstruction (LRA), macrofossil and historical data from Western Uusimaa. *J. Archaeol. Sci. Reports* **12**, 12–24 (2017).

36. Karlsen, K. B. *et al. Attersta Fossil åker, skärvstenshög och medeltida gård. Förundersökning och särskild arkeologisk undersökning Fornlämning Gällersta 39:1 Attersta 7:8 Gällersta socken Örebro kommun Närke. Stiftelsen Kulturmiljövård Rapport 2010:15*. (2013).

37. Ahlbeck, M. & Isaksson, M. *Riksväg 73 Slutundersökningar RAÄ 661, 663, 664, 665 och 666, Ösmo sn,*

Södermanland. Rapporter från Arkeologikonsult 2007:2037. (2007).

38. Guinard, M. & Stenbäck, N. *Hulje Förundersökning av en boplats från tidigneolitikum och äldre järnålder i Östergotland. RAÄ 89, Mjölby 40:5, Hulje 8:1, Skrukeby 12:5 Högby socken, Mjölby kommun, Östergötland. SAU rapport 2009:15*. (2009).

39. Hallgren, F. *Identitet i praktik. Lokala, regionala och överregionala sociala sammanhang inom nordlig trattbägarkultur.* (Coast to Coast project, 2008).

40. Edenmo, R., Graner, G., Larsson, H. & Lindholm, P. *En välordnad stenåldersboplats vid Nävertorp. UV mitt, rapport 2008:27*. (2008).

41. Hallgren, F. Rituell praktik i trattbägarkulturens norra gränsland: Tidigneolitiska gravar och offerplatser i Mälardalen, östra Mellansverige. in *Agrarsamfundenes ekspansion i nord.* (eds. Kaul, F. & Sørensen, L.) 87–101 (Nationalmuseet, 2011).

42. Larsson, L. & Broström, S. G. Meeting for transformation: A locality for ritual activities during the early neolithic funnel beaker culture in central Sweden. *Curr. Swedish Archaeol.* **19**, 183–201 (2011).

43. Edenmo, R. & Heimdahl, J. Gropkeramiskt jordbruk på Södertörn. in *Sittesta. En gropkeramisk boplats under 800 år. Arkeologi längs väg 73.* 171–210 (Riksantikvarieämbetet, 2012).

44. Lagerstedt, A. & Söderberg, M. *Russingstorp: En gård från äldre järnålder och spridda nedlslag från yngre stenålder och yngre järnålder. Fivelstad 59 och 60, Motala kommun, Östergotlad. Rapporter från Arkeologikonsult 2013:2408*. (2013).

45. Kihlstedt, B. *Boplats och gravar från tidigneolitikum vid Östra Vrå. UV mitt, rapport 2006:7.* (2006).

46. Olsson, E., Miller, U. & Risberg, J. Chapter 18 The Kyrktorp Site. in *Södertörn - interdisciplinary investigations of Stone Age sites in eastern middle Sweden. The results from the investigations for the Grödinge line inthe Södertörn peninsula.* (eds. Åkerlund, A., Olsson, E., Gustafsson, P. & Miller, U.) (Riksantikvarieämbetet, 2011).

47. Ahlbeck, M., Gill, A., Isaksson, M. & Papmehl-Dufay, L. *Jordbromalm 4:2. Arkeologisk förundersökning av stenåldersboplatsen RAÄ 233, Österhaninge sn, Södermanland. Rapporter från Arkeologikonsult 2007:2132.* (2007).

48. Runeson, H. Den goda ordningen. in *Stenålderns stationer: Arkeologi i Botniabanans spår* (eds. Gustafsson, P. & Spång, L. G.) 71–116 (Riksantikvarieämbetet, 2007).

49. Ytterberg, N. *Djurstugan: Upplands första bönder? UV GAL, rapport 2005:8*. (2005).

50. Schierbeck, A. *Hedningahällan - en undersökning för att skydda och vårda. Hälsingland Enånger socken RAÄ 68. UV Stockholm, rapport 1994:31.* (Riksantikvarieämbetet, 1994).

51. Vuorela, I. & Lempiäinen, T. Archaeobotany of the site of the oldest cereal grain find in Finland. *Ann. Bot. Fenn.* **25**, 33–45 (1988).

52. Lavento, M. Sisämaan vanhemman metallikauden väestö tutkimusongelmana. *Muinaistutkija* **4/1998**, 46–55 (1998).

53. Holmblad, P. Coastal Communities on the Move House and Polity Interaction in Southern Ostrobothnia 1500 BC - AD 1. (University of Umeå, 2010).

54. Lehtosalo-Hilander, P.-L. Dates. in *Dig It All. Papers Dedicated to Ari Siiriäinen.* (ed. Huurre, M.) 39–43 (The Archaeological Society of Finland, 1999).

55. Göransson, H. *Alvastra pile dwelling. Palaeoethnobotanical studies*. (Lund University Press, 1995).

56. Browall, H. *Alvastra pålbyggnad: 1976-1980 års utgrävningar. Västra schaktet*. (The Royal Swedish Academy of Letters, History and Antiquities, 2016).
